# Study of conformational transitions of *i*-motif DNA using time-resolved fluorescence and multivariate analysis methods

**DOI:** 10.1093/nar/gkz522

**Published:** 2019-06-14

**Authors:** Sanae Benabou, Cyril Ruckebusch, Michel Sliwa, Anna Aviñó, Ramon Eritja, Raimundo Gargallo, Anna de Juan

**Affiliations:** 1 Department of Chemical Engineering and Analytical Chemistry, University of Barcelona, Martí i Franquès 1-11, E-08028 Barcelona, Spain; 2 Univ. Lille, CNRS, UMR 8516 - LASIR - Laboratoire de Spectrochimie Infrarouge et Raman, F-59000 Lille, France; 3 Institute for Advanced Chemistry of Catalonia (IQAC), CSIC, Networking Center on Bioengineering, Biomaterials and Nanomedicine (CIBER-BBN), Jordi Girona 18-26, E-08034 Barcelona, Spain

## Abstract

Recently, the presence of *i*-motif structures at C-rich sequences in human cells and their regulatory functions have been demonstrated. Despite numerous steady-state studies on *i*-motif at neutral and slightly acidic pH, the number and nature of conformation of this biological structure are still controversial. In this work, the fluorescence lifetime of labelled molecular beacon *i*-motif-forming DNA sequences at different pH values is studied. The influence of the nature of bases at the lateral loops and the presence of a Watson–Crick-stabilized hairpin are studied by means of time-correlated single-photon counting technique. This allows characterizing the existence of several conformers for which the fluorophore has lifetimes ranging from picosecond to nanosecond. The information on the existence of different *i*-motif structures at different pH values has been obtained by the combination of classical global decay fitting of fluorescence traces, which provides lifetimes associated with the events defined by the decay of each sequence and multivariate analysis, such as principal component analysis or multivariate curve resolution based on alternating least squares. Multivariate analysis, which is seldom used for this kind of data, was crucial to explore similarities and differences of behaviour amongst the different DNA sequences and to model the presence and identity of the conformations involved in the pH range of interest. The results point that, for *i*-motif, the intrachain contact formation and its dissociation show lifetimes ten times faster than for the open form of DNA sequences. They also highlight that the presence of more than one i-motif species for certain DNA sequences according to the length of the sequence and the composition of the bases in the lateral loop.

## INTRODUCTION

DNA has been extensively studied due to its outstanding function as carrier of genetic information. The most common DNA structure found in physiological conditions is B-DNA, which is a right-handed double helical structure with Watson–Crick base pairing. However, repetitive DNA sequences have the potential to fold into non-B DNA or non-canonical structures such as hairpin, triplex, tetraplex or left-handed Z-form under certain experimental conditions. The study of these structures is of great interest because of their potential role in some diseases and aging phenomena (1). The *in vitro* formation of tetraplex structures, such as the G-quadruplex and *i*-motif structures, in the DNA sequences corresponding to the end of the telomeres and the promoter regions of several oncogenes has been demonstrated (2–4). However, the interest of DNA researchers in the *i*-motif structure decreased in past years due to the general thought that an acidic pH was necessary to stabilize the structure *in vivo*. Recently, the discovery of pH anomalies in some tumours and diseases such as amyotrophic lateral sclerosis (5,6), Huntington’s disease (7), epilepsy (8), brain tumour (9,10) and Parkinson's disease (11) triggered again this interest, especially after a recent study that provides the first direct evidence of *in vivo* presence in human cells and control regulatory functions (12).

**Figure 1.**
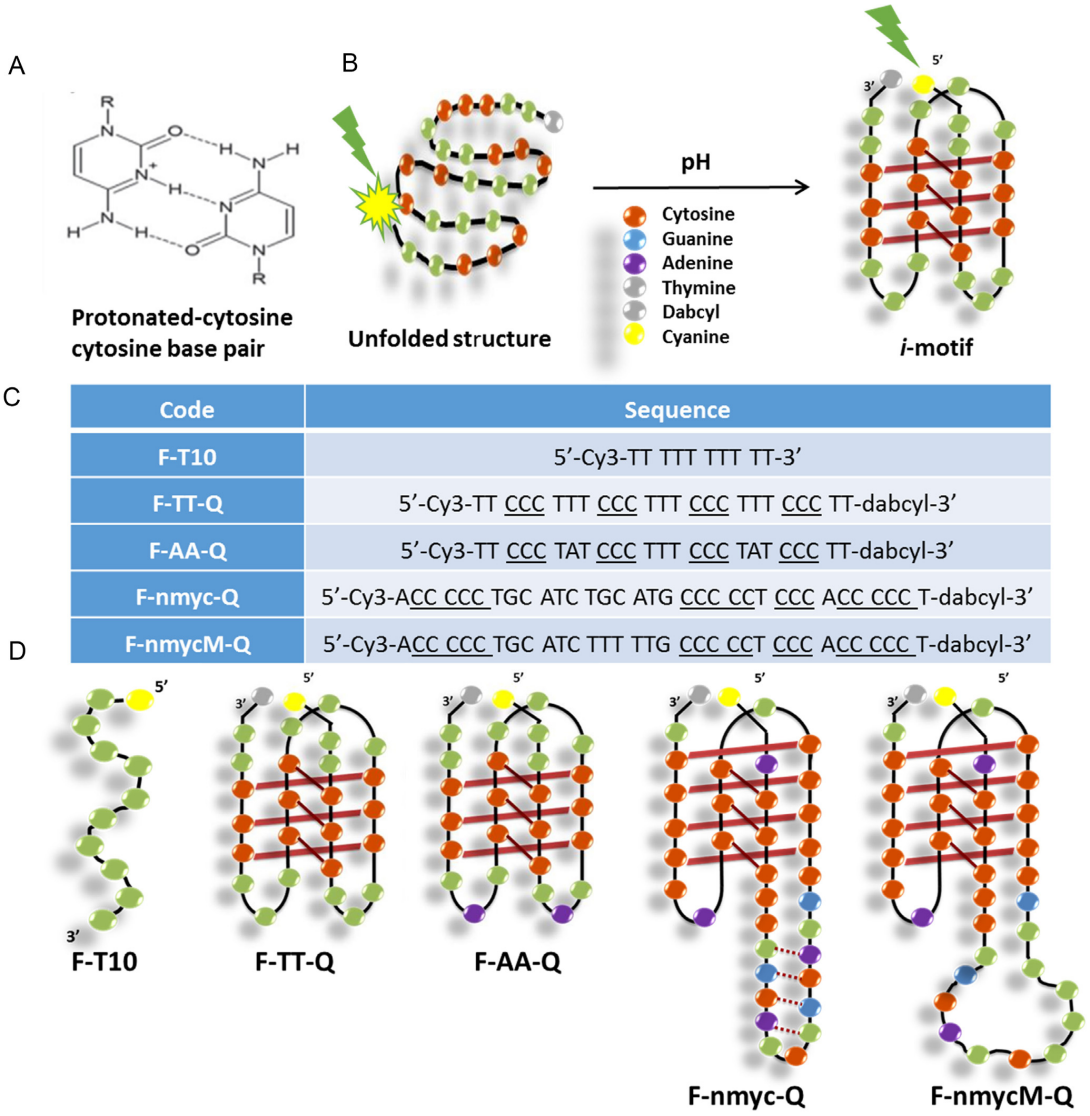
(**A**) Cytosine-protonated cytosine base pair; (**B**) Schematic process of the unfolding of the intramolecular *i*-motif structures; (**C**) Sequences studied in this work. Underlined bases are those that could be involved in the formation of C^+^ · C base pairs; (**D**) Hypothetical scheme of the intramolecular *i*-motif structures adopted by the sequences proposed to be studied.

The *i*-motif structure formed by cytosine-rich sequences at slightly acidic pH consists of parallel-stranded duplexes held together by intercalated base pairs. The formation of the C·C^+^ base pair requires the protonation of one of the cytosines at N3 (Figure 1A), the p*K*_a_ value of which is ∼4.5 (13). The formation of the *i*-motif structure in steady-state has been widely studied. The influence of variables such as pH, temperature, ionic strength and other factors such as the number of cytosine bases (14), loop length (14), molecular crowding (15–17) and the presence of ligands (18–20) on its stability has been evaluated extensively from a thermodynamic point of view. The excited state dynamics of DNA has been studied by ultrafast techniques (21–24). Recently, the *i*-motif structure has been the subject of several studies by time-resolved IR spectroscopy (25–27) as well as by time-resolved fluorescence (28–30). Fast dynamics of *i*-motif structure was reported by Choi *et al.* by using the combination of fluorescence resonance energy transfer (FRET) and fluorescence correlation spectroscopy (FCS) (28). After that, Dembska *et al.* reported steady-state and time-resolved fluorescence studies of pyrene-labeled probes based on RET gene sequence (29). However, the dynamics of processes involving *i*-motif structures has not been studied in detail.

To complete the picture of the *i*-motif behaviour, this work is oriented to study the *i*-motif conformations at different pH values, in the range between 4 and 7, in the nanosecond time scale by time-resolved fluorescence spectroscopy. To do so, several DNA sequences were labelled at the 5′ end with cyanine (a fluorophore abbreviated F) and at the 3′ end with dabcyl (a quencher abbreviated Q). When the *i*-motif is formed, the fluorophore and the quencher are very close to each other, whereas they are far away when the DNA sequence is in an unfolded open strand form (see Figure 1B).

Regarding the nature of the DNA sequences studied, two short DNA sequences (25 nucleotides), identified as F-TT-Q and F-AA-Q in Figure 1C and D, were studied. These sequences only differ in two bases (adenine or thymine) in the first and third loops (Figure 1D); thus, whereas F-AA-Q contains two opposite adenine bases, F-TT-Q contains two opposite thymine bases. The F-TT-Q sequence is considered to form a standard *i-*motif structure and will be used as a reference sequence. F-TT-Q and F-AA-Q were already reported in a previous work, performed under steady-state conditions, to investigate the influence of the nature of internal bases located at the lateral loops on the thermal and acid–base stabilities of *i*-motif structures (31). Moreover, two additional longer sequences (34 nucleotides) were studied (F-nmyc-Q and F-nmycM-Q in Figure 1C and D). The F-nmyc-Q sequence corresponds to a cytosine-rich fragment found near the promoter region of the *n-myc* oncogene (4). This gene is a member of the myc family of transcription factors. Amplification of this gene is associated with a variety of tumours, most notably neuroblastoma. The F-nmyc-Q sequence shows an unusual 12-base long loop containing two complementary TGCA sequences that could promote the formation of a stable hairpin (4). In addition, the F-nmycM-Q sequence was studied. This is a mutated version of F-nmycM-Q that does not contain G, C or A bases at the loop (because they have mutated to T) and, consequently, cannot form the proposed hairpin shown in the hypothetical scheme of F-nmyc-Q.

In a recent work, the effect of UV light on these four sequences has been studied by means of rapid-scan infrared spectroscopy and multivariate data analysis (32). The cytosine-rich sequences were exposed to UV radiation in the scale of minutes, while IR spectra were recorded and changes were resolved in the scale of milliseconds. In this way, the degradation of the DNA sequences and the formation of photoproducts upon irradiation were described. The results showed the stability of the DNA backbone and the formation of intramolecular photodimers after light irradiation periods in the millisecond scale. The extent and kinetics of the formation of those photodimers were shown to be modified by the nature of bases at the first and third loops, as well as by the presence of a lateral hairpin. In the present manuscript, however, the study carried out is completely different. Time-correlated single-photon counting (TCSPC) involves the action of a very short laser pulse that is used to bring the sequences to the excited state.The fluorescence signal is recorded in the time scale of nanoseconds. This allows monitoring the complexity of the relaxation processes, closely linked to the presence of different DNA conformations as a function of pH. Compared to steady-state spectroscopy techniques, such as circular dichroism (CD) and molecular absorption spectroscopies, TCSPC is oriented to analyse the relaxation of molecules from an excited state to a lower energy state. Through the study of the complexity of the relaxation pattern, information on the conformations of biomolecules present in the sample can be obtained.

There are different ways to exploit the information associated with TCSPC experiments, which can lead to different level of interpretation. Traditionally, TCSPC data have been analysed by global lifetime analysis (GLA) of the time-dependent fluorescence decays and sub-microsecond relaxation lifetimes associated with the different conformational events, such as the intrachain contact formation, have been obtained (33,34). Multivariate data analysis offers a new view of the TCSPC information obtained by treating simultaneously many fluorescence decays related to one or more sequences collected at different experimental conditions, e.g. pH values. In this work, principal component analysis (PCA) is used to explore the decay data and have a first overview of similarities and differences in decay behaviour among sequences monitored at different pH values. Multivariate curve resolution-alternating least squares (MCR-ALS) is more oriented to describe the number, evolution and identity of structural species found for every particular DNA sequence as a function of pH.

The results presented in the next sections will contribute to a better understanding of the changes undergone by *i*-motif DNA structures. This work also provides a general data analysis methodological framework to relate the information of the decay curves to the description of conformational transitions. To our knowledge, this is the first report about *i*-motif conformational transitions as a function of pH monitored by time-resolved fluorescence spectroscopy and interpreted by using the combination of the global decay fitting and multivariate analysis methods.

## MATERIALS AND METHODS

### Experimental section

The oligonucleotides studied were prepared on an Applied Biosystems 394 synthesizer by solid-phase 2-cyanoethylphosphoroamidite chemistry at a 0.2 μM scale. A 3′-dabcyl solid support, (1-(4,4′-Dimethoxytrityloxy)-3-[O-(N-4′-carboxy-4-(dimethylamino)-azobenzene)-3-aminopropyl))-propyl-2-O-succinoyl-lcaa-CPG, Link Technologies, USA) was used and, in the last step, the oligonucleotides were modified with cyanine dye using Cyanine-3-CE Phosphoramidite (Cyanine 540), Link Technologies). After the standard ammonia treatment (NH_3_, 55°C, 6 h), the oligonucleotides were desalted with a Sephadex column, analysed by HPLC on a semipreparative column X-Bridge™ OST C_18_ (10 × 50 mm, 2.5 μm). The mobile phase was formed by solution A (5% acetonitrile, ACN, in 0.1 M aqueous triethylammonium acetate, TEAA) and solution B (70% ACN in 0.1 M aqueous TEAA). A 10 min linear gradient from 0% to 30% of B, flow rate 2 ml min^−1^ was used for the separation. The identity of the synthesized sequences was confirmed by matrix-assisted laser desorption ionization time-of-flight (MALDI-TOF) spectral detection.

The molecular beacon strategy was applied to increase the intensity of DNA fluorescence for the present study. To do so, the DNA sequences TT, AA, n-myc and n-mycM) were labelled at the 5′ and 3′ ends with cyanine as a fluorophore (F) and dabcyl as a quencher (Q), respectively (Figure 1D). When the DNA sequence is unfolded, the fluorophore is far from the quencher and an intense fluorescence signal is observed; instead, when the sequence forms the *i*-motif, the fluorophore and the quencher are close and a large decrease of fluorescence is noticed (see Figure 1B). In addition, a polythymidine sequence labelled at 5′ end with cyanine (F-T10) was used as a control sequence because it cannot form any folded structure. Samples for measurements were prepared in 20 mM phosphate buffer with 150 mM KCl and the pH was adjusted pH with 2 M citric acid solution. CD spectra were recorded on a Jasco J-810 spectropolarimeter equipped with a Julabo F-25/HD temperature control unit and using Hellma quartz cells (10 mm path length, 3000 μl volume) covering the spectral range of 220–360 nm. CD measurements were used to confirm that no structural change was induced by labelling. In addition, steady state absorption and fluorescence emission spectra were also recorded. Absorbance spectra were recorded on an Agilent 8453 diode array spectrophotometer using Hellma quartz cells (10 mm path length, 3000 μl volume), and the spectral range covered was 400–700 nm. Fluorescence emission spectra were recorded on an Aminco Bowman AB-2 fluorimeter. Emission spectra were recorded between 530 and 650 nm, and fluorescence intensities were recorded every 1 nm. The excitation wavelength was set to 544 nm, the photomultiplier voltage to 520 V, and the excitation and emission band pass to 4 nm.

All the fluorescence decays used in this work have been recorded with the TCSPC technique. Briefly, Cyanine-3 was excited with a 544 nm laser pulse obtained by a Ti:sapphire laser (Coherent Chameleon Ultra II, 80 MHz, 200 fs)) coupled with an intracavity frequency doubled OPO (APE) and a pulse picker (4 MHz). Fluorescence signal acquisition was performed using a FT200 PicoQuant spectrometer. Emission wavelengths were selected with a Czerny-Turner type monochromator equipped with 0.5 mm slits providing a 4 nm spectral resolution. Fluorescence was detected with a cooled microchannel plate (Hamamatsu R3809U) photomultiplier tube and recorded with a TimeHarp260 (pico model, bin time of 25 ps bin) TCSPC system. The instrument response function (IRF) was obtained by analysing the scattering signal of the 544 nm beam scattered in a Ludox solution. The full width at half maximum (FWHM) of the IRF was equal to 50 ps, which can considered the time-resolution of our setup. The fluorescence decays were measured through an emission polarizer set at the magic angle (versus excitation polarizer) and acquisition was stopped when the maximum of events reached a value of 3 × 10^4^ counts. The traces were recorded at seven emission wavelengths (560, 565, 570, 575, 580, 590 and 610 nm) for each DNA sequence and at pH values 7.0, 6.0, 5.0 and 4.0, except for the control sequence F-T10, where traces were recorded only at three emission wavelengths: 565, 580 and 610 nm.

### Data analysis

Three different data analysis methods have been used in this work to analyse the experimental fluorescence decay traces: GLA, PCA and MCR-ALS. GLA methods approximate experimental data by a discrete sum-of-exponentials function. On the other hand, both PCA and MCR-ALS are soft-modelling methods that are not based on the compliance of any model based on rate constants. PCA has been used to observe similarities and dissimilarities among fluorescence decay traces measured for different sequences and pH values. As shown below, PCA does not take into account any chemical or physical information during the mathematical analysis. MCR-ALS, instead, is oriented to describe the number and the evolution of structural species found for every particular DNA sequence as a function of pH. The method uses information on natural properties of decay traces and concentration values within the process modelling task. Below, a more detailed description on the data analysis approaches used is provided.

### Global fitting analysis

TSPC decays are traditionally described as following the model in Equation (1), which is based on a sum of exponential terms, related to components or relaxation events, convoluted with an instrumental response function (IRF):

(1) \begin{equation*}I ( t ) = \mathop \int \nolimits_{ - \infty }^t {\rm IRF} \left( {t^{\prime}} \right) \mathop \sum \nolimits_{i = 1}^n {A_i} \ {e^{ - \frac{{t - t^{\prime}}}{{{\tau _i}}}}}\ {\rm d}t^{\prime}\end{equation*}

where *I* is the vector of intensity (counts) data, *A_i_* is the amplitude of the *i*th component, in counts, at time zero and *τ_i_* is the lifetime of the *i*th component. IRF is the measured instrumental response function of the instrument.

GLA fits the model represented in Equation (1) to several decays collected on the same chemical system. The lifetimes are estimated globally whereas the pre-exponential amplitudes are adjusted for each trace. The calculation consists first of an iterative reconvolution of the instrument response (IRF) with the exponential components. Fitting error is then minimized using a non-linear least-squares approach abased on the Levenberg–Marquardt algorithm (35). Standard exponential (up to four exponential terms) and multimodal lifetime distribution models can be fitted to the recorded data. The quality of the fit is assessed by the value of *χ*^2^ (below 1.1), by the absence of systematic trends on the weighted residuals

(2) \begin{equation*}\left(R(i) = 1/ {\sqrt {{\rm Decay}}} (i)\,[{\rm Decay}(i) - {\rm Fit}(i)] \right)\end{equation*}

versus time as well as by the random character of the residual autocorrelation functions. Reduced *χ*^2^, plot of weighted residuals and their autocorrelation function are available to facilitate the assessment of the fit quality and to help in the selection of the proper number of exponential terms to be included in the fitting task. In this work, for a particular DNA sequence and pH value, every set seven-wavelengths decays is fitted globally to obtain the related lifetimes. GLA performed using the FluoFit software (PicoQuant; version 4.6.6).

The calculation of the fluorescence average lifetime ($\tau$) with amplitude weighted was done by following this equation (${\boldsymbol{\bar{\tau }}} = \mathop \sum \nolimits^ \tau \times A/100$).

### Data structure for PCA and MCR-ALS analysis

**Figure 2.**
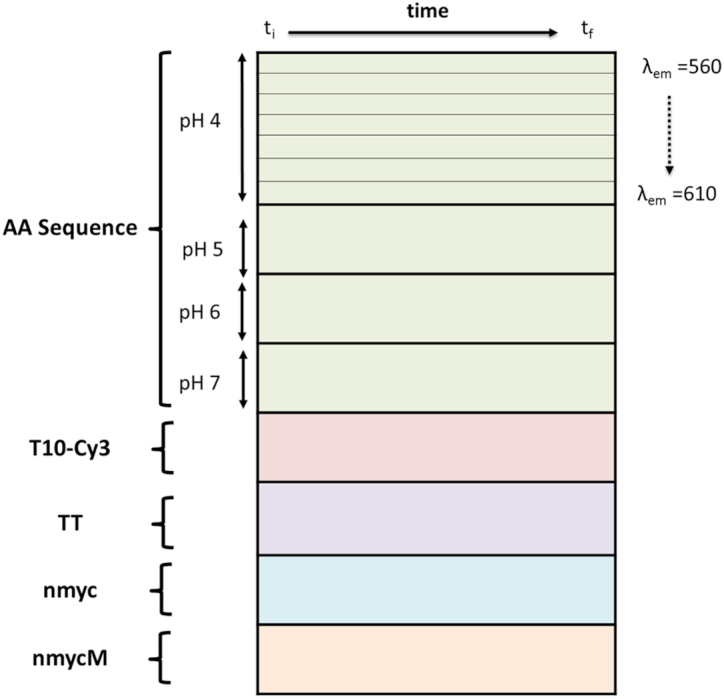
Scheme of data structure for PCA analysis.

All the experimental fluorescence decays were organized in a data matrix **D** (*m × n*), where each row contains the measured curve at one emission wavelength and at one pH value (Figure 2). In other words, every row in data matrix **D** corresponds to the decay in Equation (1). The *n* columns correspond to the *n* time values at which the counts were recorded. The decays were ordered in the rows as a function of pH, from pH 4 to 7. In the case of PCA analysis, the decays of all DNA sequences acquired at the different pH values were organized in a single data matrix, according to the scheme in Figure 2. Instead, MCR-ALS was performed separately on the decays of each particular sequence acquired in the full pH range of interest.

### Principal component analysis (PCA)

PCA is a multivariate analysis technique that allows explaining most of the information contained in a data matrix **D**, like the one in Figure 2, with a small number of orthogonal components (36). In general, PCA decomposes a data matrix **D**, sized (*m* x *n*) according to Equation (3):

(3) \begin{equation*}{{\bf D}}\ = {\rm{\ }}{{\bf T}}\ {{{\bf P}}^{{\bf T}}} + {\rm{\ }}{{\bf E}}\end{equation*}

Here, **T** and **P^T^** are called, respectively, the scores and loadings matrices (37). The dimensions of matrices **T** and **P^T^** are *m* x *s* and *s* x *n*, respectively, where *s* is the number of principal components needed to describe properly the chemically relevant variance of the original data matrix **D**. The matrix **E** (*m* x *n*) contains the residual information unexplained by the PCA model. This mathematical decomposition is carried out under constraints of maximum explained variance and orthogonality. In PCA, the *s* components are ordered based on the variance explained in descending order. Therefore, the first component explains the largest percentage of variance in matrix **D**. The second component is orthogonal to the first one and explains the largest percentage of variance not described by the first component and so forth. In this work, the score plots allow displaying each row in the dataset, related to a decay curve, and, as a consequence, help in finding similarities and dissimilarities amongst the dynamic behaviour of the considered DNA sequences at the different pH values, whereas the loadings provide qualitative information about the relevant decay curve features to differentiate amongst DNA sequences.

Prior to PCA, the data were preprocessed by autoscaling, which uses column mean-centring followed by division of column standard deviation.

### Multivariate curve resolution alternating least squares (MCR-ALS)

Multivariate curve resolution alternating least squares (MCR-ALS) (38–40) is an iterative self-modelling approach that optimizes **C** and **S^T^** under constraints and has been successfully applied in numerous fields (41–43). MCR-ALS has been widely used in process analysis because it provides pure component spectra and process profiles by using the information coming from the mixture spectra recorded during the evolution of the chemical system (44,45). Notably, MCR-ALS has been extensively used to analyse data from photochemical processes studied at very short time scales and dedicated data preprocessing, data configurations and constraints have been developed to deal with the specificities of this kind of measurements (46).

In the context of the present study, the objective of MCR-ALS analysis is to gain a better understanding of the conformational transitions involved in the formation of *i*-motif structure in each sequence at the different pH values when these structures are studied in a short time scale, out of steady-state conditions. The use of MCR-ALS to unravel the evolution of the conformations involved in the *i*-motif formation as a function of pH lies in the fact that every particular conformation can be defined by a basic decay trace, and the evolution of all decay traces measured experimentally must be a concentration-weighted sum of the basic decay traces at a particular pH. In mathematical terms, this means that the matrix **D** of fluorescence decay traces for a particular DNA sequence collected at the different pH values may be described by assuming a bilinear decomposition, as shown in Equation (4):

(4) \begin{equation*}{{\bf D}}\ = {\rm{\ }}{{\bf C}}\ {{{\bf S}}^{{\bf T}}} + {\rm{\ }}{{\bf E}}\end{equation*}

For time-resolved spectroscopic data, **D** (*m × n*) contains the *m* measured fluorescence decays (at *n* counts) ordered as a function of pH. **S^T^** (*p × n*) contains the related basic fluorescence decays of each of the *p* DNA conformations involved in the *i*-motif formation at short time scale and **C** (*m × p*) is formed by columns describing the evolution of the relative contribution of each particular DNA conformation along the process as a function of pH. **E** (*m × n*) is the matrix containing the variance unexplained by the model. It should be pointed out that the basic profiles (concentration profiles and pure decay curves) associated with each of the conformations considered in MCR-ALS are different from the scores and loadings calculated in PCA. This is because the profiles provided by MCR-ALS fulfil constraints that reflect physical and/or chemical meaning, whereas the scores and loadings in PCA are subject to the mathematical constraint of orthogonality. This is why **C** and **S^T^** matrices calculated with MCR-ALS are different from **U** and **V^T^** matrices calculated with PCA.

A good advantage of MCR-ALS is that no prior or little information is needed about the nature and composition of the compounds involved in the process of interest. MCR-ALS aims at resolving the bilinear model shown in Equation (3) by using the sole information contained in the raw dataset **D**. The MCR-ALS *modus operandi* can be summarized in the following steps:

1. Determination of the number of components in the raw dataset **D**;
2. Generation of initial estimates of either the evolving concentration profiles, **C**, or spectra, **S^T^**;
3. Iterative least-squares calculation of the spectra matrix, **S^T^**, and the matrix of concentration profiles, **C**, under constraints until convergence is achieved (i.e. the difference of lack of fit, LOF, between two consecutive iterations is below a threshold or a predefined number of iterations is reached).

The percentage of lack of fit (LOF) and explained variance (*R*^2^) are used to determine the fit quality of the resolution model and are calculated by using (Equations 5 and 6), respectively:

(5) \begin{equation*}{\rm LOF} = \sqrt {\frac{{{{\sum}_i}{{\sum}_j}{{\left( {{{ d}_{i,j\ - }}\skew{6}\hat {\ {d}}i,j} \right)}^2}}}{{{{\sum}_i}{{\sum}_j}\ {d}_{i,j}^2}}} \ \end{equation*}

(6) \begin{equation*}{R^2} = \frac{{{{\sum}_i}{{\sum}_j}\,\skew{6}\hat {d}_{i,j}^2}}{{{{\sum}_i}{{\sum}_j}\ d_{i,j}^2}}\ \end{equation*}

where *d_i,j_* and $\skew{6}\hat{d}$_i,j_ are the *ij*th element of **D** and the *ij*th element of the reconstructed matrix by the MCR-ALS model, respectively.

A more detailed description of the algorithm can be found elsewhere (39,44,45,47). Briefly, the number of components can be estimated from singular value decomposition (SVD). It can be seen that the procedure needs an initial estimate of either **C** or **S^T^**. Here, the initial **C** matrix was generated by using evolving factor analysis (EFA) (48), which is a local rank analysis method (49), very appropriate to obtain initial estimates of concentration profiles from sequential evolutionary processes. EFA analysis is suitable for this study because the conformations related to *i*-motif formation are known to evolve in a sequential manner as a function of pH, i.e. the decay of one conformation gives rise to the formation of the next and there are no reversals in this evolution. The resolved profiles in **C** and **S^T^** by MCR-ALS may not be unique, being subject to rotational ambiguities. Constraints are therefore used to limit the number of possible solutions and to provide chemically meaningful concentration profiles and resolved pure decay traces (48,50,51). In this study, non-negativity was applied to the concentration profiles and to the pure decay traces in **S^T^**, since both kinds of profiles are known to be formed by positive values.

As we commented above, from the MCR-ALS analysis we can get two matrices **C** and **S^T^**. In order to know better the lifetimes associated with the pure resolved decays, the pure decay traces obtained from MCR-ALS analysis were fitted to the minimum number of exponential terms by means of the curve fitting tool (cftool) of Matlab without considering the contribution of the IRF, which could not be exported from the instrument. Fitting error is then minimized using an algorithm based on a non-linear least-squares approach. The quality of the fit in the models is assessed by the value of *R*^2^ (over 0.999) and by testing the significance of each fitted exponential term comparing the value of the lifetime fitted with the associated error through a *t*-test.

## RESULTS AND DISCUSSION

### Steady state experiments

In this work, four different sequences that have been functionalized at the 5′ end with Cyanine-3 and at the 3′-end with dabcyl were studied (see Figure 1 and Supplementary Figure S1 for details in the labelled structure). First and foremost, CD measurements were conducted to elucidate whether the DNA structure is affected by the covalent attachment of the dyes. Supplementary Figure S2 in the supplementary material shows the CD spectra of the labelled and unlabelled sequences at pH 4.0. Those spectra are similar before and after labelling and show the characteristic spectral features of the *i*-motif structure (16,52). These results clearly indicate that the DNA structure remains intact after labelling with the dyes.

**Figure 3.**
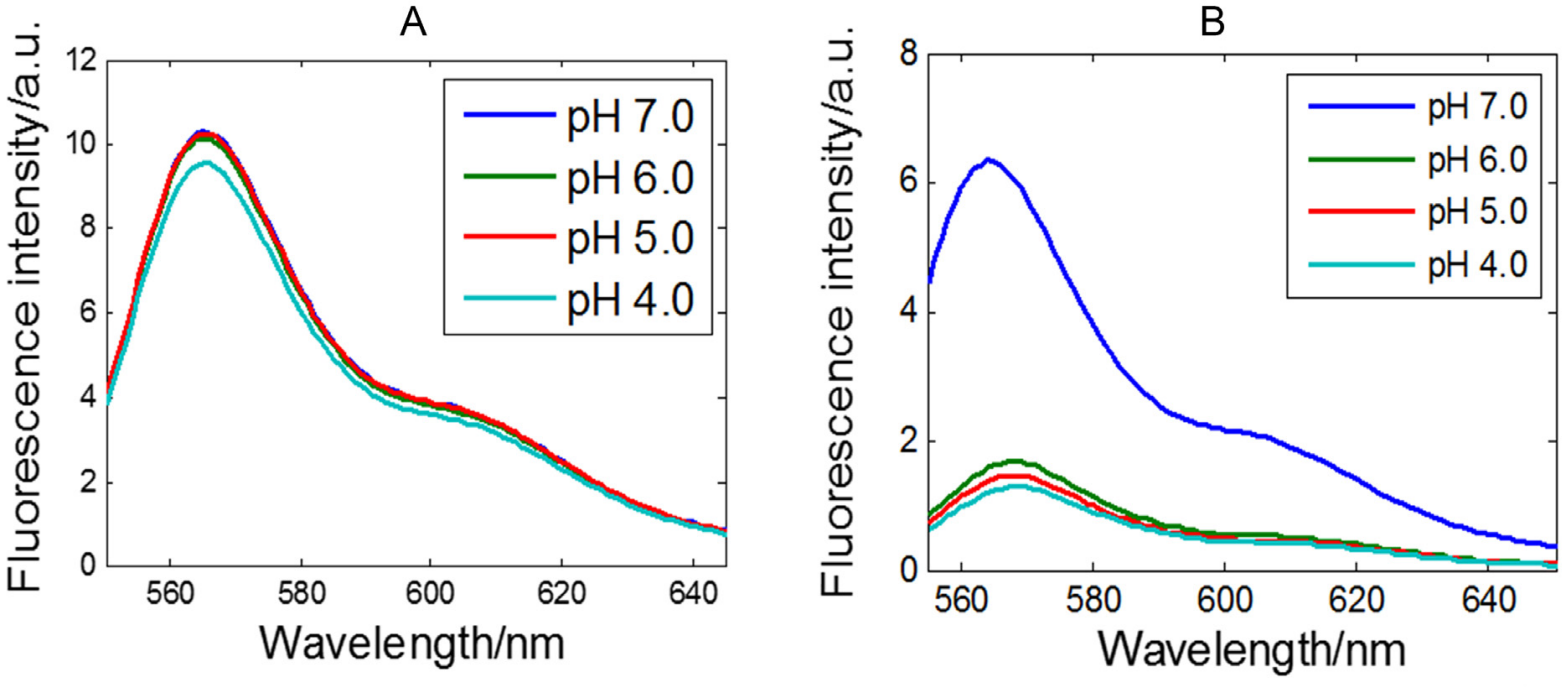
Fluorescence emission spectra (excitation 540 nm) of F-T10 (**A**) and F-TT-Q (**B**) sequences in solutions at different pH values. DNA concentration: 1 μM at 25°C.

Supplementary Figure S3 shows steady state absorbance and emission spectra at different pH for all sequences. As an example of the general behaviour of the DNA sequences studied, Figure 3 shows the fluorescence spectra of the F-T10 and F-TT-Q sequences at different pH values. It may be observed that the fluorescence intensity of cyanine is reduced upon decreasing pH for the F-TT-Q sequence, whereas high fluorescence intensity is preserved for F-T10 in the pH range from 7 to 4 (only a very slight decrease can be seen at pH 4, likely due to small differences of stacking amongst T bases). It is known that fluorescence intensity of cyanine-3 does not suffer changes in the pH working range because its p*K*_a_ value is 9.7 (53), as can be seen with the high fluorescence intensities in the sequence F-T10. Therefore, the decrease of fluorescence with decreasing pH in the F-TT-Q sequence is closely related to the folding of the sequence to yield the *i*-motif structure, the stability of which is higher at pH 4.5. That is, upon decreasing pH, the open structure of F-TT-Q is folded to form the *i*-motif with a tetrameric structure, resulting in a sufficient decrease of the distance between the fluorophore and quencher that induces a drop in the fluorescence signal intensity. Steady state absorbance and emission spectra at different pH for all sequences can be seen in Supplementary Figure S3.

### Global decay fitting

First of all, the fluorescence average lifetimes (${\boldsymbol{\bar{\tau }}}$) of all sequences studied at different pH were calculated (Table 1). Table 1 shows similar average lifetimes of the F-T10 control sequence at different pH, which indicate that no significant conformational changes take place as a function of pH. Such a conclusion was expected since F-T10 is a model sequence for unfolded DNA structure. In all other sequences, there is a clear decrease of the average lifetime between samples at pH 7 and at more acidic pH values, which can be associated with a conformational change related to the formation of *i*-motif structure. Observing the evolution within the pH range between pH 6 and 4, the average lifetime decreases when pH decreases for short sequences F-TT-Q and F-AA-Q, whereas there is no clear tendency of variation of average lifetime with pH for long sequences F-nmyc-Q and F-nmycM-Q.

**Table 1.** Fluorescence average lifetime ($\bar{\tau }$) of all sequences studied at different pH

	$\bar{\tau }$ (Amplitude weighted)/ns			
Sample\pH	7	6	5	4
F-T10	0.67	0.72	0.73	0.72
F-TT-Q	1.45	0.30	0.18	0.11
F-AA-Q	1.46	0.26	0.20	0.11
F-nmyc-Q	2.03	0.17	0.14	0.18
F-nmycM-Q	1.13	0.16	0.16	0.13

In order to further examine the conformational dynamics of F-TT-Q, F-AA-Q, F-nmyc-Q and F-nmycM-Q sequences in open form (single-stranded structure) and presenting *i*-motif conformation, global decay fitting as described in the data analysis section has been performed. In every global fitting, all decay traces recorded for the same sequence at a particular pH value were analysed together and a single set of lifetimes was obtained.

**Figure 4.**
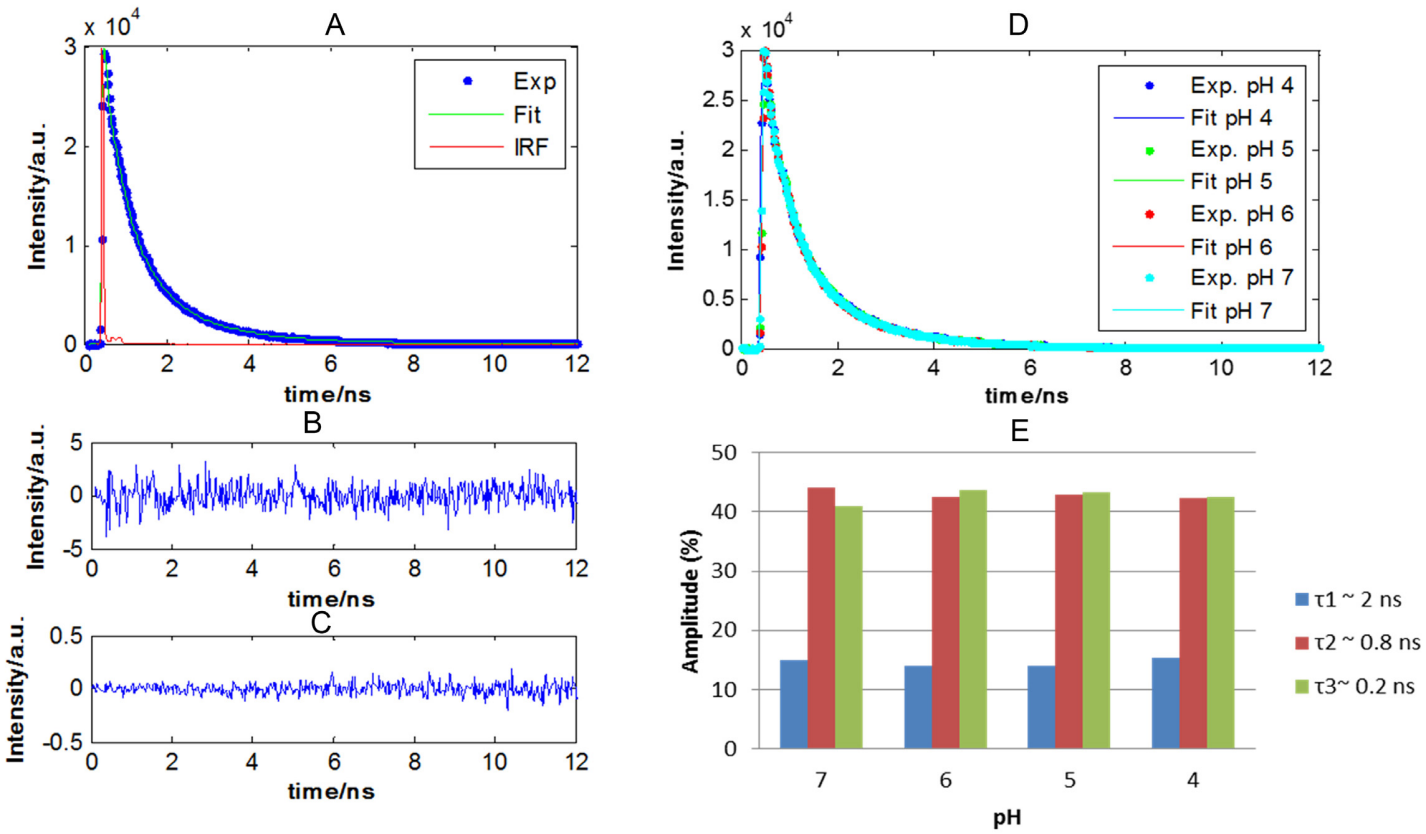
Results of the mathematical fitting of the decay traces of F-T10 sequence. (**A**) Raw fluorescence decay, fitted decay and IRF decay at pH 5.0. (**B**) Residuals. (**C**) Autocorrelation of residuals. (**D**) Raw and fitted data of fluorescence decay profiles as a function of pH. (**E**) Distribution of the fractional amplitude of each lifetime contribution as a function of the pH of the control sequence F-T10.

First of all, to evaluate pH-dependent contributions to the decays that could potentially come from the fluorophore, the fluorescence decays of the control sequence F-T10 at the different pH values were analysed. All decay profiles from F-T10 could be fitted by a triexponential function convolved by the IRF with lifetimes of ∼1.88, 0.80 and 0.20 ns with slight difference around these values depending on the sequence (see Supplementary Table S1). Figure 4A shows an example of a raw fluorescence decay of F-T10 at pH 5.0 with the fitted decay and the IRF contribution. Figure 4B and C show the fitting residuals and their autocorrelation function, respectively.

Figure 4D shows all fluorescence decay profiles of the fluorophore dye (Cyanine-3) of F-T10 measured at different pH values and the fitted curve coming from global decay fitting. Figure 4E shows the contributions (amplitudes) of the different lifetime components to the experimental decay curve.

First, it can be observed that the shape of the decay traces recorded for the control sequence F-T10 is practically independent of pH. The fluorescence decays for this DNA single strand were fitted to the minimum number of exponential terms that produced randomly distributed residuals. The control sequence sample presented a long (2 ns) component that accounts for 40% of the decay. The other two lifetimes (0.80 and 0.20) fitted for F-T10 are in very good agreement with values previously published for a similar sequence (54). Sabanayagam *et al.* also reported TCSPC lifetime measurements of Cy3 conjugated to DNA (55). In this work, the authors interpreted the non-monoexponential behaviour of the decay as due to an equilibrium between two conformational states of the control sequence where the dye senses two environments of different polarity. But, in another study reported by Sanborn *et al.*, the results showed that Cy3 has the highest activation energy for isomerization when attached to the 5′ terminus of single-stranded DNA (54). Thus, the presence of a long-lived component in the fluorescence decay of these samples suggests that a fraction of the Cy3 molecules interacts with the DNA strand so that the fluorophore experiences a high barrier for isomerization. To add to the previous hypotheses, Norman *et al.* proposed that Cy3 is stacked to the end of the double helix, based on NMR results (56). Hence, a possible model that would be consistent with our results involves the existence of three populations. In a first population (time constant 2 ns), Cy3 would interact strongly with the DNA strand (stacking interaction). In the other two populations (0.2 and 0.8 ns), the interaction would be disrupted and isomerization would occur with a lower activation energy upon excitation, and *cis*–*trans* isomerization of C = C bonds in the dye polymethine chain competes with fluorescence emission for deactivation of the molecule from the excited to the ground state (54–56). The shorter time constant is similar to lifetime of Cy3 in water.

For all DNA sequences at pH 7, three components are required to fit the data and lifetimes similar to the ones of the control sequence were obtained (see Supplementary Tables S2 and S3), which could be an indication of a similar single-stranded DNA structure. In contrast, all fluorescence decays for sequences at pH 4, 5 and 6 need to consider the sum of four exponentials, which point out to the presence of a different conformation at this pH range, presumably the *i-*motif.

**Table 2.** Fluorescence decay fitting parameters of F-TT-Q, F-AA-Q, F-nmyc-Q and F-nmycM-Q sequences, life time (*τ*) and the % of fractional amplitude

(A) Global decay analysis (FluoFit)										
Sample	F-TT-Q			F-AA-Q			F-nmyc-Q		F-nmycM-Q	
pH	7	6&5	4	7	6&5	4	7	6&5&4	7	6&5&4
*τ*_1_(ns)	2.34(7) ^$^	3.03(1)	3.01(1)	2.38(7)	3.05(1)	3.00(2)	2.62(5)	2.69(1)	2.52(5)	2.68(1)
% Amplitude	38.17	4.96	1.75	37.94	4.72	1.64	64.06	3.39	56.00	3.03
*τ*_2_(ns)	1.11(8)	0.85(2)	0.88(1)	1.13(8)	0.94(2)	0.88(2)	1.12(1)	0.88(1)	1.06(1)	0.89(2)
% Amplitude	44.09	4.17	2.59	43.72	4.06	2.58	29.39	3.26	32.63	2.86
*τ*_3_(ns)	0.35(2)	0.18(4)	0.18(3)	0.34(2)	0.17(5)	0.18(5)	0.24(4)	0.17(4)	0.27(3)	0.17(4)
% Amplitude	17.75	13.87	7.93	18.34	12.90	7.73	6.56	10.20	11.38	10.68
*τ*_4_(ns)		0.03(7)	0.03(2)		0.03(3)	0.03(2)		0.03(2)		0.03(4)
% Amplitude		77.01	87.73		78.33	88.05		83.16		83.43
(B) Pure traces from MCR-ALS analysis (cftool)										
Sample	F-TT-Q			F-AA-Q			F-nmyc-Q		F-nmycM-Q	
pH	7*	6&5*	4*	7*	6&5*	4*	7*	6&5&4*	7*	6&5&4*
*τ*_1_(ns)	2.8 (2)^$^	3.08(3)	2.9(2)	2.63(3)	2.66(3)	2.8(1)	2.69(2)	2.54(4)	2.61(2)	2.52(4)
% Amplitude	25	20	14	48	18	12	68	18	69	18
*τ*_2_(ns)	1.33(5)	0.45(1)	0.60(5)	0.46(1)	0.469(9)	0.56(4)	1.23(3)	0.49(2)	1.11(2)	0.48(2)
% Amplitude	75	28	29	52	42	28	32	30	31	28
*τ*_3_(ns)		0.065(1)	0.089(4)		0.048(1)	0.073(3)		0.065(1)		0.066(1)
% Amplitude		66	67		40	60		52		53

(A) Global decay analysis by FluoFit and (B) fitting of pure resolved traces by MCR-ALS analysis by cftool. *Fitting is done on the pure traces that could be associated with this pH range. ^$^: Number in parentheses is the first significant figure of error.

**Figure 5.**
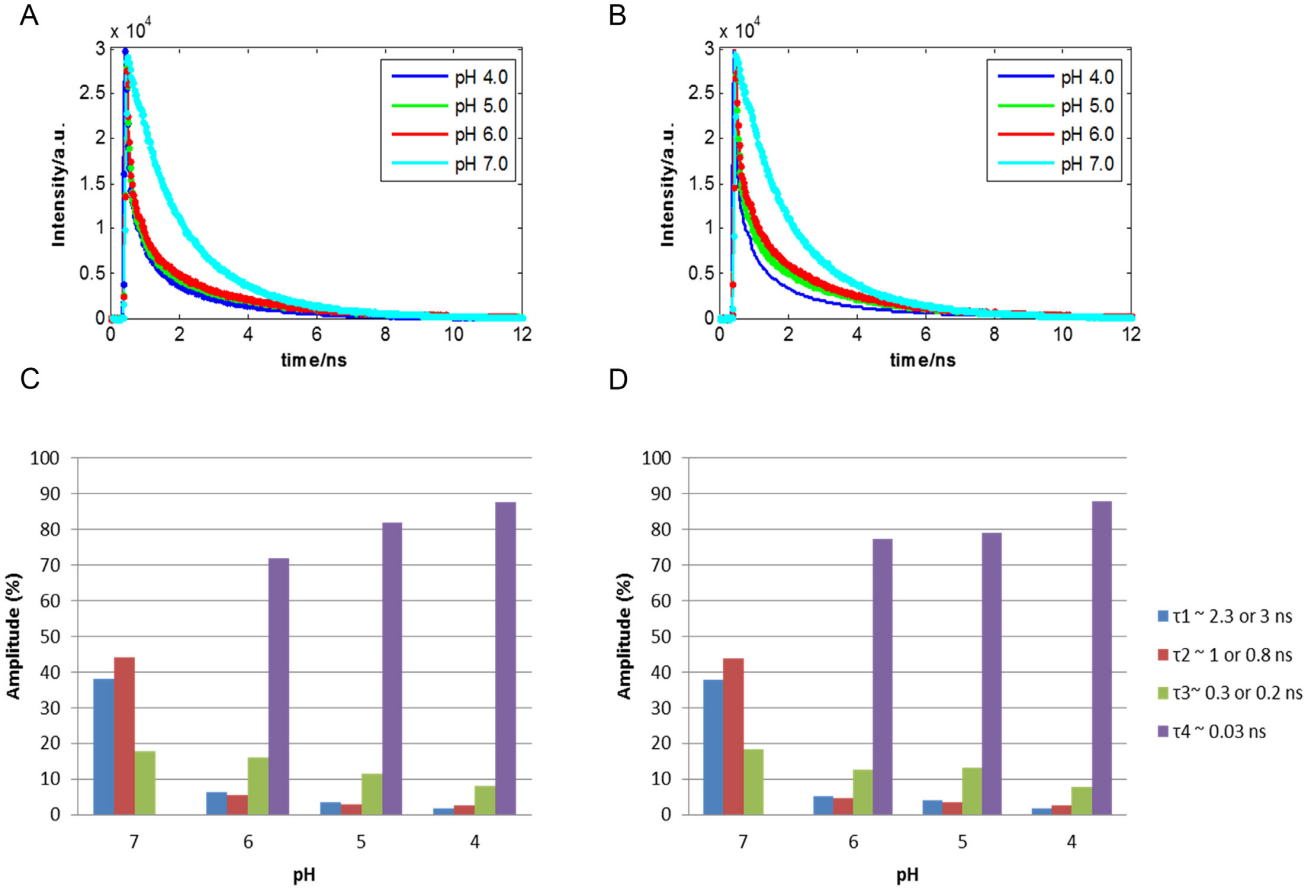
Raw and fitted traces at different pH of F-TT-Q (**A**) and F-AA-Q (**B**) sequences. Distribution of the fractional amplitude of each lifetime with the pH of the F-TT-Q (**C**) and F-AA-Q (**D**) sequences.

The decay profiles of F-TT-Q and F-AA-Q sequences at acidic pH, i.e. in the pH range from 4 to 6, shown in Supplementary Table S1 were fitted with a four-term exponential function. A global fitting analysis of all decay profiles of each particular sequence at each pH revealed four lifetimes of ∼0.03, 0.2, 0.8 and 3 ns. As shown in Figure 5C and D, the fast component (*τ*_4_ = 0.03 ns) is dominant and present only at acidic solutions, whereas three components (*τ*_1_ ≈ 3 or 2.3 ns, *τ*_2_ ≈ 1.0 and *τ*_3_ ≈ 0.3 ns) are present in the whole pH range studied.

Considering the expectable presence of *i*-motif and the open form in the sequences studied, two decay components should be observed in the time-resolved fluorescence experiments for F-AA-Q and F-TT-Q sequences. However, as mentioned above, we could observe four components with different fluorescence lifetimes, indicating that, at least, four species are involved in the relaxation from the excited state in the pH range studied. On one hand, the decay time of the fast components (*τ*_4_ = 0.03 ns) in acidic pH solutions was attributable to a folded structure that could induce the fast quenching from the fluorophore to the quencher due to close proximity. Considering the properties and necessary conditions for *i*-motif formation, we suggest that the fast-decay component of 0.03 ns observed at acidic pH can be attributed to the *i*-motif, whereas the other three components may be attributed to the stacking interaction of the Cy3-DNA and to the isomerization, as it already happened with the control sequence. Figure 5C and D shows also that the contribution of the *i*-motif component increases when pH decreases, being the highest at pH 4. Even the lifetime for this component decreases slightly when going from pH 6 to 4. These facts could point out to some differences in the *i*-motif structure when the pH gets lower and the formation of the structure becomes easier. Finally, the distribution of components is very similar in F-TT-Q and F-AA-Q sequences, which would mean that the nature of the bases in the central loop does not have a strong influence on the fluorescence characteristic times of cyanine attached at the 5′ end of these sequences. On the other hand, we suggest that although the fluorescence lifetime of the slow-decay component observed at pH 7 (tau1 = 2.6 ns) is shorter than the corresponding lifetime at acidic pH (3.0 ns), the former is attributed to the open form. The reason for the decrease of the fluorescence lifetime of the fluorophore molecule from 3 to 2.6 ns when going to higher pH values is probably due to the fact that the rigid structure of *i*-motif has less degrees of freedom and no radiative relaxation pathway decrease and, thus, the fluorescence lifetime is longer.

**Figure 6.**
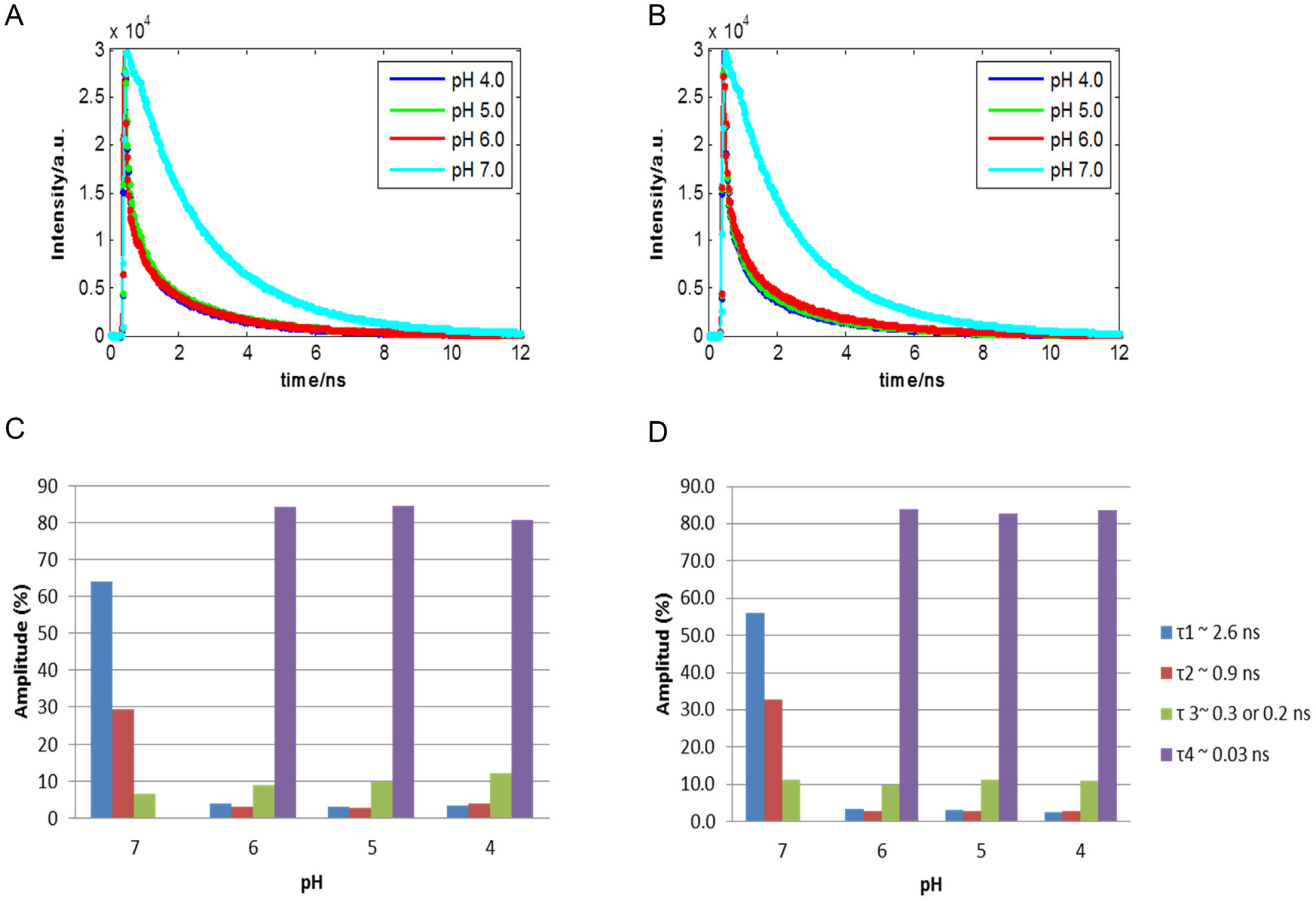
Raw and fitted traces at different pH of the F-nmyc-Q (**A**) and F-nmycM-Q (**B**) sequences. Distribution of the fractional amplitude of each lifetime with the pH of the F-nmyc-Q (**C**) and F-nmycM-Q (**D**) sequences.

Decay traces related to the long DNA sequences F-nmyc-Q and F-nmycM-Q at acidic pH were also fitted using a sum of four exponential terms. The global fitting analysis of the decay profiles of each acidic pH revealed four lifetimes approximately equal to 0.03, 0.3, 0.9 and 2.6 ns in the light-induced excitation of F-nmyc-Q and F-nmycM-Q (Supplementary Table S2). As shown in Figure 6, the fast component (*τ*_4_ = 0.03 ns) is present only at acidic solutions and it is similar to the one observed by F-TT-Q and F-AA-Q sequences, whereas three components (*τ*_1_ = 2.6 ns, *τ*_2_ = 0.9 and *τ*_3_ = 0.3 ns) are present in the whole pH range studied.

As in case of the short sequences, the fast component (*τ*_4_ = 0.03 ns) was attributed to a folded *i*-motif structure that can induce the fast quenching from the fluorophore to the quencher due to close proximity. In contrast to the F-TT-Q and F-AA-Q sequences, Figure 6C and D showed that the lifetimes and contribution of the fast component related to the *i*-motif is approximately constant at the different pH values, which could mean that there is no much structural variation of this motif in the pH range studied. Finally, the lifetimes obtained and the distribution of the related components are very similar in F-nmyc-Q and F-nmycM-Q sequences, which would mean that the presence of the hairpin in the loop does not have a strong influence on the fluorescence curve decay of cyanine attached at the 5′ end and, therefore, on the *i*-motif structure of these two sequences.

### Principal component analysis

**Figure 7.**
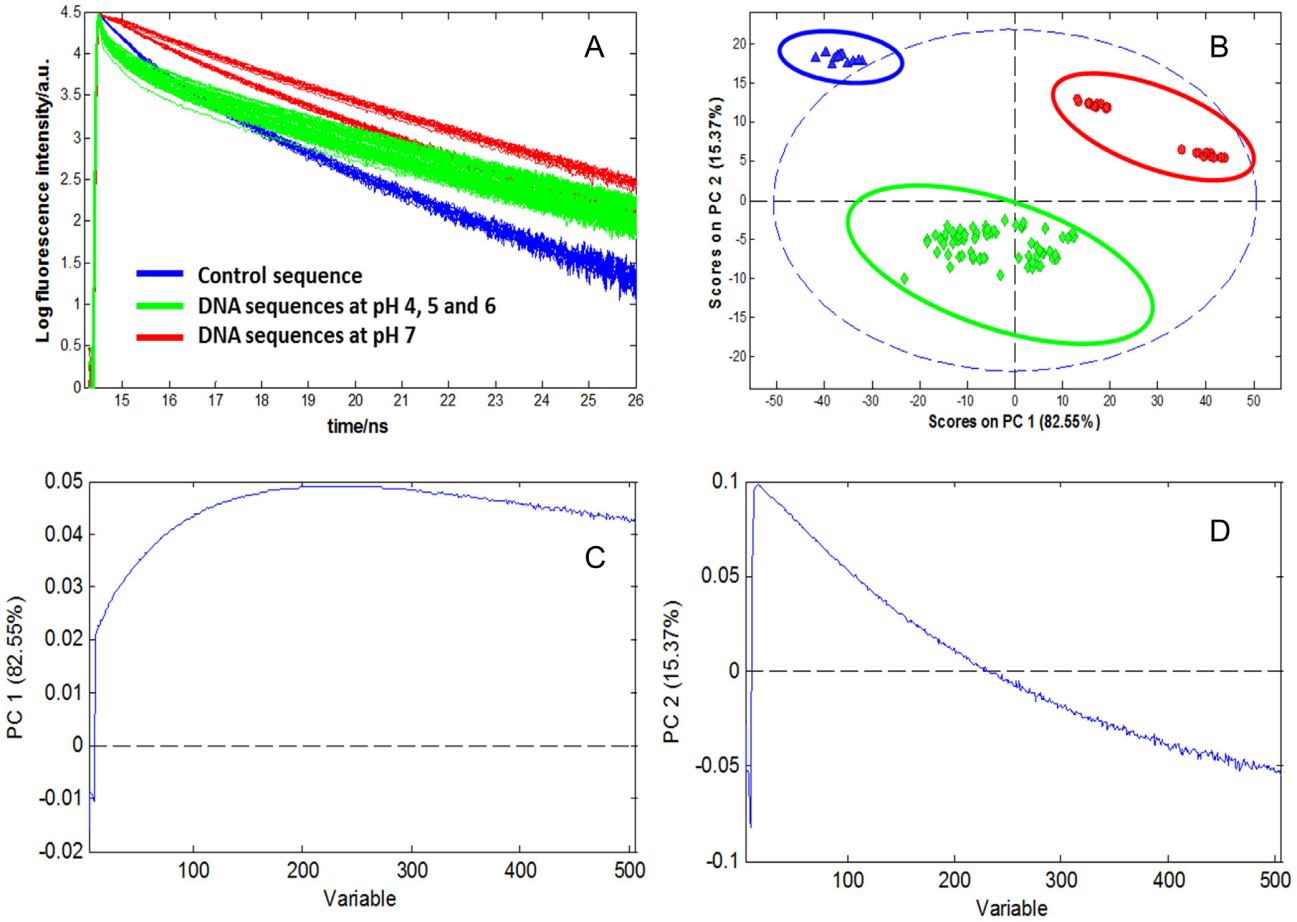
Fluorescence decay traces of DNA sequences and related PCA results. (**A**) Raw data of fluorescence decays of control (F-T_10_) and DNA sequences (F-T10, F-TT-Q, F-AA-Q, F-nmyc-Q and F-nmycM-Q) at different pH values. (**B**) Scores plot, (**C**) Loading of PC1 and (**D**) Loading of PC2.

First of all, PCA has been carried out on the data matrix **D**, and the results are displayed in Figure 7. These results provide a first insight on the similarities and differences of dynamic behaviour of all the DNA sequences studied in this work. The raw data, as well as the PCA calculated scores and loading plots are shown in Figure 7. Every dot in the score plot represents a decay curve from a particular sequence at a certain pH value and emission wavelength. Sequences with similar behaviour are clustered in the score plot. Loadings are abstract traces related to decay curve features relevant to describe main trends and differences amongst sequences.

The PCA model needed two principal components (i.e. equal to 2) to explain 97% of the total variance of matrix **D**, distributed between first and second component (PC1 and PC2) as 82% and 15%, respectively. The score plot of PC2 versus PC1 (Figure 7B) allowed distinguishing three clusters or groups of DNA sequences with similar behaviour: (i) the control sequence at pH 4–7 (blue circle), (ii) the sequences F-AA-Q, F-TT-Q, F-nmyc-Q and F-nmycM-Q sequences at pH 7 (red circle) and (iii) the sequences F-AA-Q, F-TT-Q, F-nmyc-Q and F-nmycM-Q at pH 4–6 (green circle). The behaviour of the control sequence is different from the rest of the samples and it is independent of pH since sequences in the whole pH range are close together, which means that they have a similar relaxation behaviour. Thus, PC1 separates fundamentally the control sequence from the rest of sequences, while PC2 separates the control sequence and sequences at pH 7 from sequences at pH 4, 5 and 6. Looking at PC2, it can be observed that the control sequence and the sequences at pH 7 have similar behaviour, whereas sequences at more acidic pH values are clustered and well separated from the rest. This is related to the fact that the conformation of cytosine-rich sequences is strongly dependent on pH and at least two different conformations, related to the unfolded and folded *i*-motif structures, are expected to be present at the pH range studied (4,31). Thus, the sequences at acidic pH are separated from the others, and this can be attributed to the formation of the folded structure of *i*-motif. By contrast, the control sequence, known to be unable to form the *i*-motif, and sequences at pH 7, where the open strand form is the expected conformation, group together.

**Figure 8.**
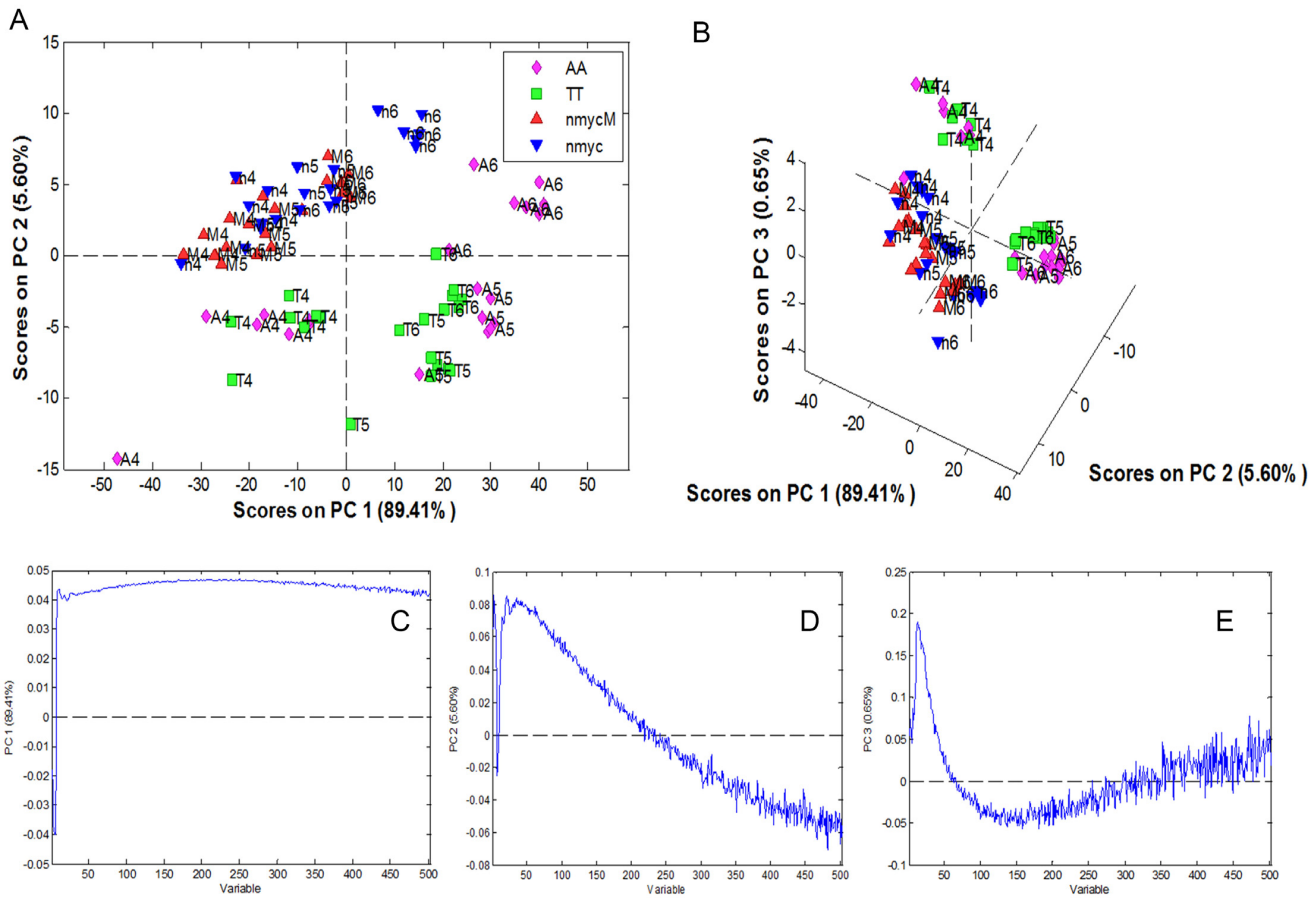
Local PCA of F-TT-Q, F-AA-Q, F-nmyc-Q and F-nmycM-Q sequences at pH 6, 5 and 4 classifying by sequences. (**A**) Scores of PC1 versus PC2, (**B**) Scores of PC1 versus PC2 versus PC3, (**C**) Loading of PC1, (**D**) Loading of PC2 and (**E**) Loading of PC3.

Once these most different clusters were detected, a local PCA model for the group related to the sequences at pH 4, 5 and 6 was done to highlight the differences or similarities amongst sequences that form the *i*-motif structure. Figure 8 shows the corresponding scores and loading plots.

The results of local PCA showed that the DNA sequences clustered in three different groups. On the one hand, short (F-TT-Q and F-AA-Q) and long sequences (F-nmyc-Q and F-nmycM-Q) are clearly separated from each other, which could be related to the fact that the *i*-motif structures in longer sequences have an additional C-C^+^ base pair. However, the scores corresponding to longer sequences F-nmyc-Q and F-nmycM-Q show no clear pattern related to the variation of pH between 4 and 6. Instead, scores corresponding to short F-AA-Q and F-TT-Q sequences form two clearly separated clusters, one associated with sequences at pH 4 and another with sequences at pH 5 and 6. This could suggest two different conformations of *i*-motif structure in short sequences. Within the long sequences, the observation of a single group could be related to the presence of a single *i*-motif structure. These conclusions match well with the results obtained applying global decay fitting, where almost no differences in contribution and lifetime for the fastest *i*-motif component could be detected in sequences F-nmyc-Q and F-nmycM-Q, whereas a decrease of lifetime with pH and a difference in the contribution of the fastest *i*-motif component was seen in the shortest sequences F-TT-Q and F-AA-Q, which could be related to a higher complexity in the *i*-motif contributions. Nevertheless, neither PCA nor global fitting is a conclusive technique to prove or discard the presence of one or more *i*-motif species in DNA sequences. To test this hypothesis, additional analysis was carried out by MCR-ALS.

### Multivariate curve resolution-alternating least squares

**Figure 9.**
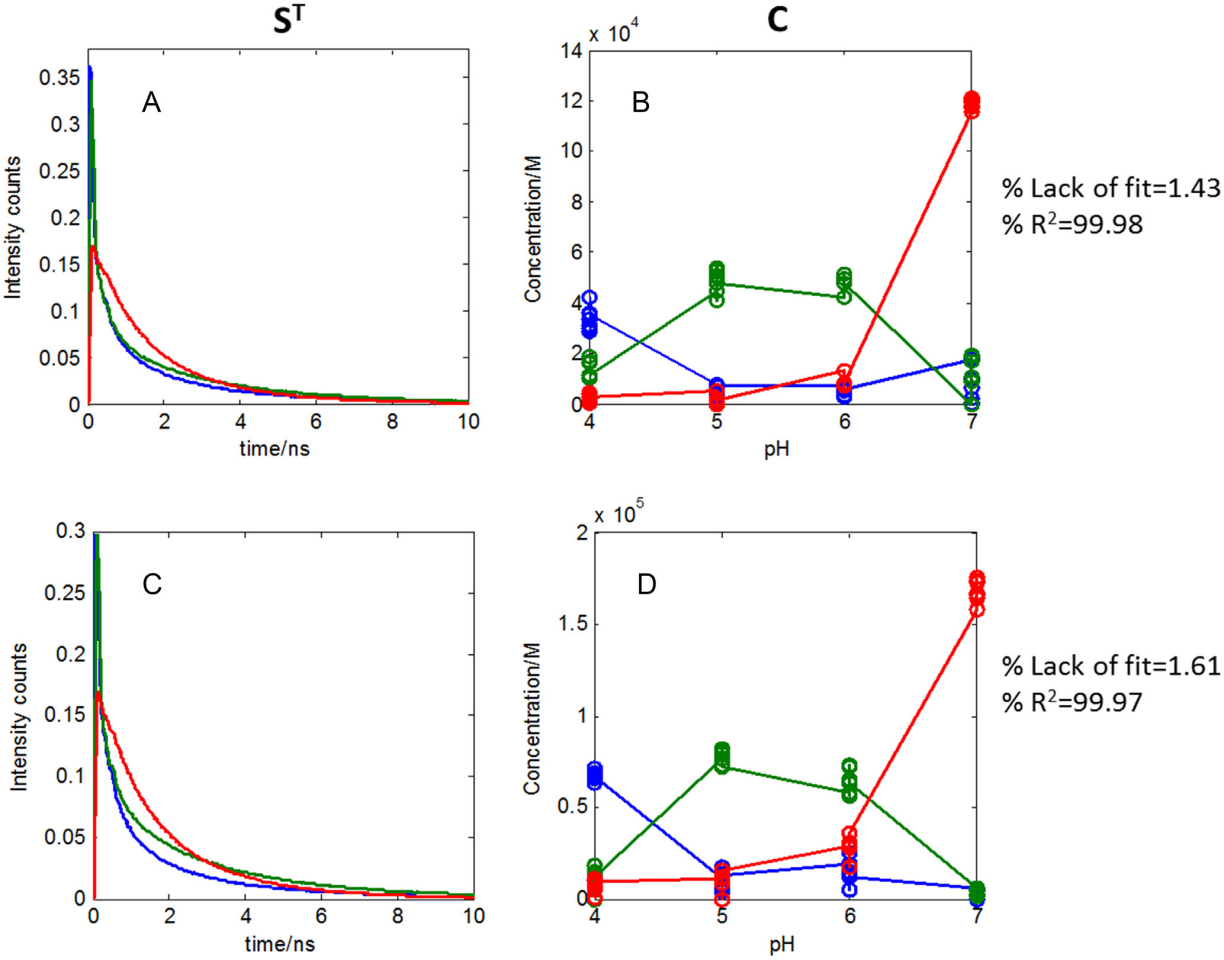
MCR-ALS analysis of the decays of F-TT-Q and F-AA-Q sequences. (**A**) Pure spectra of F-TT-Q, (**B**) Concentration profiles of F-TT-Q, (**C**) Pure spectra of F-AA-Q and (**D**) Concentration profiles of F-AA-Q. Every point in concentration profiles represents the scatter in concentration at a certain pH and emission wavelength for the different decay traces acquired in the same experimental conditions.

To gain a better understanding of the nature of the conformations involved in the formation process of *i*-motif structure as a function of pH when studied at short time scale, MCR-ALS was applied separately to each DNA sequence by analysing all the decay traces collected at the different pH values. MCR-ALS provides as a result the concentration profiles (**C**) and basic decay traces related to each sequence conformation (**S^T^**). The MCR-ALS analysis of the traces at different pH of each sequence was performed applying the constraints of non-negativity to **C** and **S^T^**. In this case, starting values for the evolving profiles of each species were calculated by means of EFA. The results obtained for the F-TT-Q and F-AA-Q sequences at different pH values are presented in Figure 9.

The decays of F-TT-Q and F-AA-Q sequences were successfully modelled with three components. The three species would correspond to a folded structure of *i*-motif dominant at pH 4 (blue in Figure 9), a different folded structure of *i*-motif dominant at pH 5 and 6 (in green in Figure 9) and a final species related to an unfolded structure of *i*-motif (in red in Figure 9). This result is an example of the usefulness of multivariate analysis and, particularly, MCR-ALS, to elucidate the presence and characteristics of two contributions of *i*-motif structure, a difficult task from the sole inspection of the data or from the lifetimes obtained by global fitting approach. This behaviour also agrees with the PCA results, where sequences of F-AA-Q and F-TT-Q were distributed in two separate groups in the score plot according to the pH, one with sequences at pH 4 and one at pH 5–6.

**Figure 10.**
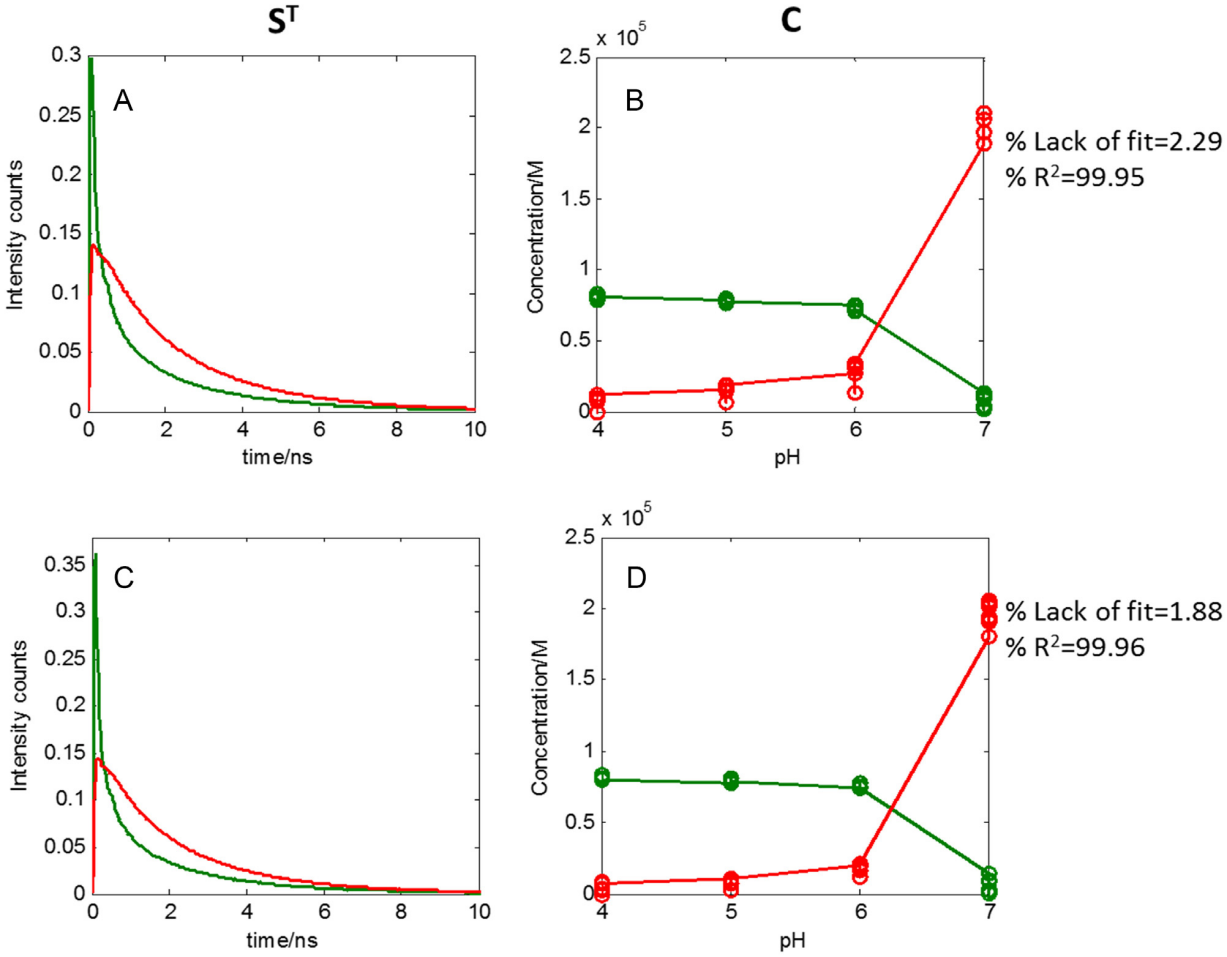
MCR-ALS analysis of the decays of F-nmyc-Q and F-nmycM-Q sequences. (**A**) Pure spectra of F-TT-Q, (**B**) Concentration profiles of F-TT-Q, (**C**) Pure spectra of F-AA-Q and (**D**) Concentration profiles of F-AA-Q.

The MCR-ALS results obtained for the F-nmyc-Q and F-nmycM-Q sequences are presented in Figure 10.

For long sequences, F-nmyc-Q and F-nmycM-Q, the decays were successfully modelled with two components. The first species would correspond to the folded structure of *i*-motif from pH 4 to 6 (in green in Figure 10) and the second species to the unfolded structure of *i*-motif at pH 7 (in red in Figure 10). Again, this conclusion matches the PCA results, where these sequences at pH values between 4 and 6 appeared in a single compact group.

In previous melting steady-state studies at different pH values on the same melting sequences, the results have shown that the melting transition occurs in two phases, involving three different DNA conformations. In the case of short sequences F-TT-Q and F-AA-Q, more than one species is present at 25°C (temperature of the time-resolved fluorescence measurements), whereas the long sequences F-nmyc-Q and F-nmycM-Q showed only one dominant species at the same temperature, because the global melting process happened at higher temperatures. Therefore, the complex melting behaviour of these sequences matches the results obtained by MCR-ALS in this work that point to two *i*-motif species for short sequences, linked to the two conformations present at 25°C, and only one for longer sequences, linked to the presence of a sole *i*-motif in the same working conditions.

Our results showing the possible presence of more than one *i*-motif conformation for some of the sequences studied in the experimental conditions used are in tune with those presented by Dhakal *et al.*, who found also the coexistence of the partially folded form and *i*-motif in the C-rich human insulin linked polymorphism region (ILPR) oligonucleotides using the laser tweezers technique (57). They also suggested that the formation of *i*-motif is decreased by increasing pH, while the partially folded structure can be found at pH between 5.5 and 7.0. Moreover, using experimental and theoretical methods, Dettler *et al.* reported that the classical *i*-motif structure is predominant at slightly acidic pH (pH 4.2–5.2), whereas another *i*-motif-like structure is the most significant species closer to neutral pH (58). After that, Choi *et al.* reported that the fast dynamics of *i*-motif with a compact tetraplex is due to the intrinsic conformational changes at the fluorescent site, including the motion of the alkyl chain connecting the dye to DNA, whereas the slow intrachain contact formation observed from the open form is due to the DNA motion, corresponding to an early stage interaction in the folding process of the unstructured open form. These conclusions are partially supported by our results and confirm that the fast-decay components of characteristic times around 0.03 ns can be attributed to *i*-motif species.

Finally, in order to complete the study by MCR-ALS and to know better the lifetimes associated with the pure resolved decays, the pure decay traces obtained from MCR-ALS analysis were fitted to the minimum number of exponential terms (see Supplementary Figure S4). For an easier comparison, the results of this fitting (Table 2B) are shown together with those provided by classical global decay analysis when the raw decay curves of the sequences were fitted at the different pH values.

First of all, a general decrease of one exponential contribution is seen in the fitting of all pure resolved traces obtained by MCR-ALS by cftool (Table 2B) when compared with the results provided by classical global decay fitting by Fluofit (Table 2A). However, it has to be considered that cftool fitting is performed without considering the effect of the IRF function and removing some few initial time channels so that the behaviour of all measured traces is considered from the moment that the decay tendency starts. This has generally resulted in a decrease of one exponential contribution in the fit of all sequences with respect to the results obtained by Fluofit, where all time frame is considered as well as the action of IRF. If fitting is performed on the raw decay curves by cftool without considering IRF, the same number of exponential contributions as in Table 2B are found (see Supplementary Table S3).

Focussing on the cftool analysis of the pure resolved traces of all DNA sequences by MCR-ALS (Table 2B), we found that, at the most, three components with different fluorescence lifetimes are involved when sequences are studied in the full pH range 4–7. In addition, it is also observed that the component with lowest lifetime is lost at pH 7, which confirms the absence of *i*-motif conformations in this experimental condition.

The decay profiles of all sequences at acidic pH were fitted with three exponential functions. A curve fitting analysis of all pure decay profiles of each particular sequence at each pH revealed three lifetimes of ∼0.06, 0.5 and 2.5 ns, similar in magnitude to the global decay results presented in Table 2A. As shown in Table 2B, the fast component (*τ*_4_ = 0.06 ns) is present only at acidic solutions, indicating the presence of *i*-motif conformations, whereas two additional components (*τ*_1_ ≈ 2.5 ns and *τ*_2_ ≈ 1.0 or 0.5) are present in the whole pH range studied.

## CONCLUSIONS

In this work, intramolecular folding dynamics of *i*-motif DNA were studied by combining time-resolved fluorescence, global fitting approach and multivariate methods, such as PCA and MCR-ALS. Most of the differences observed for the transitions that the *i*-motif structure undergoes in different sequences that were seen in experiments carried out from pH 4.0 to 6.0 in steady state. In light of the previous studies, an understanding on the short conformational dynamics of the structural change of cytosine-rich sequences that could form *i*-motif structures was necessary to complement previous results.

First of all, using TCSPC analysis, it could be shown that the intrachain contact formation and dissociation for *i*-motif is 10 times faster than that for the open form when comparing the lifetime of these structures. The fast dynamics of *i*-motif with a compact tetraplex is due to the intrinsic conformational changes at the fluorescent site, whereas the slow intrachain contact formation observed from the open form and the folded structure of *i*-motif is due to the DNA motion, corresponding to the stacking interaction of the Cy3-DNA and to the isomerization. Moreover, MCR-ALS has allowed differentiating two conformations of *i*-motif structure in the case of the short sequences, meanwhile only one was detected in long sequences, indicating that the length of DNA sequences and the number of C·C^+^ pairs affect the conformational dynamics of *i*-motif structures. This conclusion matches the PCA results, where short sequences presenting *i*-motif appeared distributed in two groups and the long sequences in one compact group and the differences in lifetime behaviour of the *i*-motif contribution as obtained by global decay fitting. Additional factors such as the difference of bases in the lateral loop of sequences or the possibility to form additional structural elements like hairpins did not seem to affect significantly the dynamics of folding of *i*-motif structures at short time scales.

The application of the three data analysis approaches has been useful due to the valuable and complementary information that can be extracted from each one. PCA yielded an overview on differences and similarities of sequence behaviour associated with the sequence structure or due to pH variations. From the global decay fitting, the lifetimes associated with each sequence at the different pH values were accurately determined, taking into account the influence of the IRF. These lifetimes were informing about the different events sensed by the fluorophore, linked or not to the *i*-motif formation, although no definitive conclusions could be inferred about the number and evolution of DNA conformations as a function of pH. Instead, MCR-ALS provided additional information about the presence and number of the different conformations associated with the pH evolution and characterized each of these species with a basic decay curve. The curve fitting of the basic decay curves provided by MCR helped to extract good approximate lifetimes related to each DNA conformation. In conclusion, the combination of MCR-ALS followed by the curve fitting tool of resolved decay curves can be a good alternative to provide information on the evolution of the species involved in the formation of *i*-motif structures as a function of pH and to extract the lifetimes of the pure conformations.

The results presented support previous studies by TCSPC, which suggested faster lifetimes for folded *i*-motif conformations when compared to open forms, and confirmed the presence of more than one *i*-motif species for certain DNA sequences. Our results can be easily extended from these *in vitro* studies to data obtained from the analysis of human cells. In addition these structures can be therapeutic targets in cancer or other pathological conditions, and it may provide useful insights on the relationship between structure and their biological roles in genome.

As an additional outcome of this work, MCR-ALS has been proven for the first time to be a powerful tool to study the conformational changes of *i*-motif structures, monitored by time-resolved fluorescence. The combination of MCR-ALS and femtosecond fluorescence allows differentiating the conformations associated with the *i*-motif structures and helps in the detection of potential intermediates. Furthermore, the posterior fitting of the resolved decay curves allows the characterization of the lifetimes of pure conformations related to the *i*-motif formation. This new data analysis strategy can be generally applied to any biological or chemical process monitored by TCSPC that involves transitions among different species. Future improvements can be envisioned that could involve the use of IRF, when available, in the resolved decay curve fitting step, or the inclusion of decay curve fitting with IRF convolution within the MCR-ALS optimization process.

## DATA AVAILABILITY

MCR-ALS graphic interface is freely available on the web at http://www.mcrals.info/.

## Supplementary Material

gkz522_Supplemental_File
