# Supplementary material for: Study of conformational transitions of i-motif DNA using time-resolved fluorescence and multivariate analysis methods
Source: Nucleic Acids Res. 2019 Jun 14;47(13):6590–605. doi: 10.1093/nar/gkz522 (PMC6649798; doi:10.1093/nar/gkz522)
Supplement: gkz522_Supplemental_File [file gkz522_supplemental_file.pdf]

Supplementary Information

Study of conformational transitions of i-Motif DNA by femtosecond fluorescence and MCR-ALS methods

Sanae Benabou[1], Cyril Ruckebusch [2], Michel Sliwa[2], Anna Aviñó[3], Ramon Eritja[3], Raimundo Gargallo[1], Anna de Juan[1]

[1] Department of Chemical Engineering and Analytical Chemistry, University of Barcelona, Martí i Franquès 1-11, E-08028 Barcelona, Spain

[2] Université de Lille Sciences et Technologies, LASIR CNRS, Cité Scientifique, 59655 Villeneuve d'Ascq, France.

[3] Institute for Advanced Chemistry of Catalonia (IQAC), CSIC, Networking Center on Bioengineering, Biomaterials and Nanomedicine (CIBER-BBN), Jordi Girona 18-26, E-08034 Barcelona, Spain

Contents

Section	Page
Supplementary Figure 1. Chemical structure of Cy3 and Dabcyl	2
Supplementary Figure 2. CD spectra	2
Supplementary Figure 3. Absorption and emission spectra	3
Supplementary tables 1 and 2. Fluorescence decay fitting parameters	5
Supplementary Figure 4. Curve fitting analysis of pure traces	6
Supplementary table 3. Comparison of fitting on pure resolved traces by MCR-ALS and on raw experimental decay curves (cftool)	7

Supplementary Figure 1. Chemical structure of the 5’-CY3 and 3’-DABCYL modifications

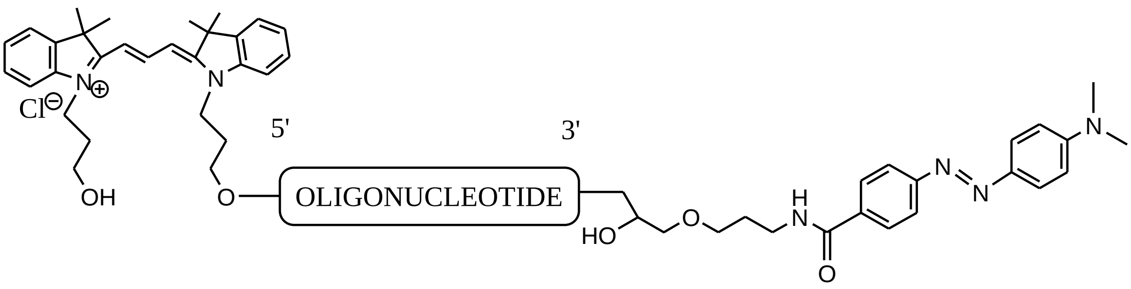

CYANINE 3 DABCYL

Supplementary Figure 2. CD spectra

CD spectra of the unmodified (Blue) and 5’-Cy3, 3’-DABCYL-modified (red) TT, AA, nmyc and nmycM sequences at pH 4.

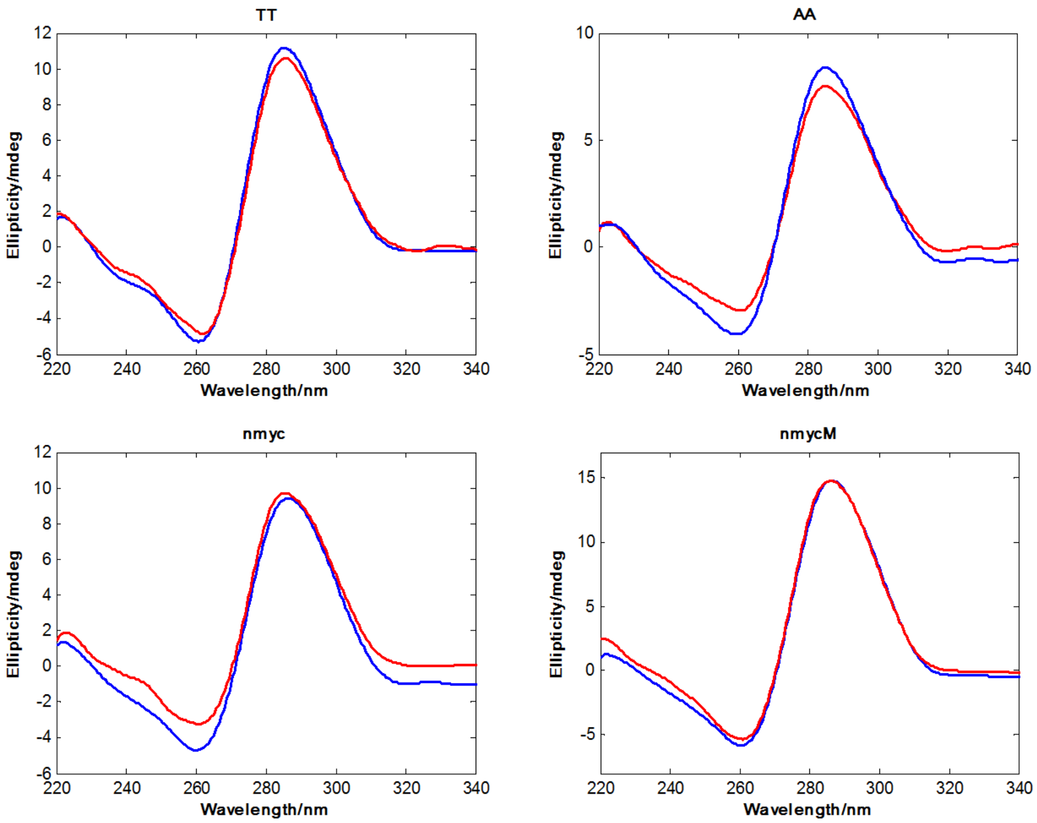

Supplementary Figure 3. Absorption and emission spectra

Absorption and emission spectra at different pH for the fluorophore (Cy3) and all sequences studied. Conditions: 20mM phosphate buffer, 150mM KCl and 25°C

<table>
<tr><th>Sequence</th><th>Absorption spectra</th><th>Emission spectra</th></tr>
<tr><td>Cy3</td><td>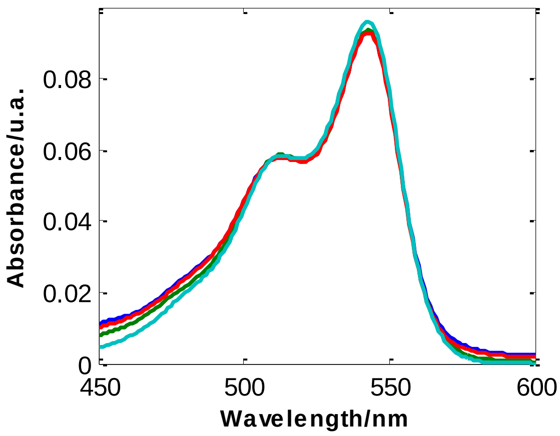
</td><td>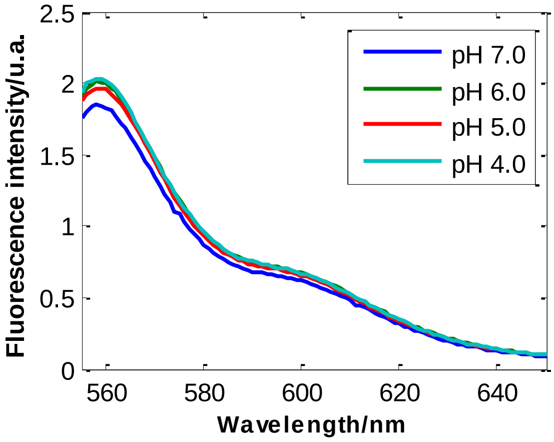
</td></tr>
<tr><td>F-T10</td><td>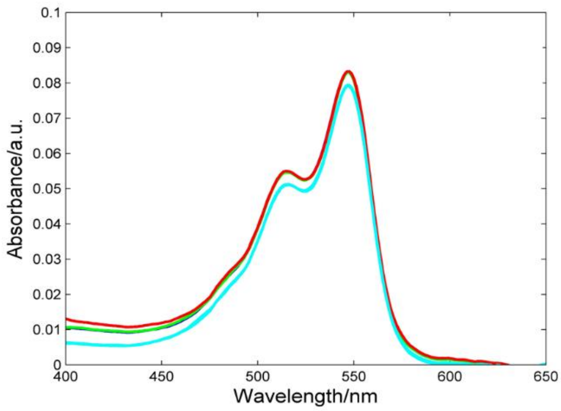
</td><td>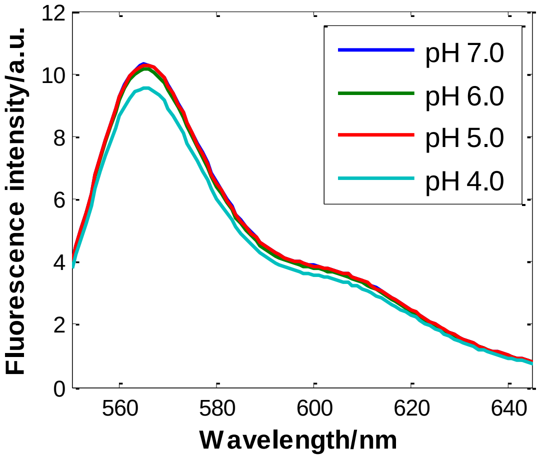
</td></tr>
<tr><td>F-TT-Q</td><td>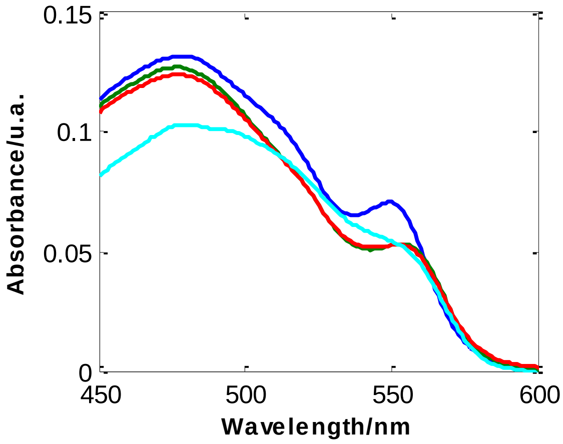
</td><td>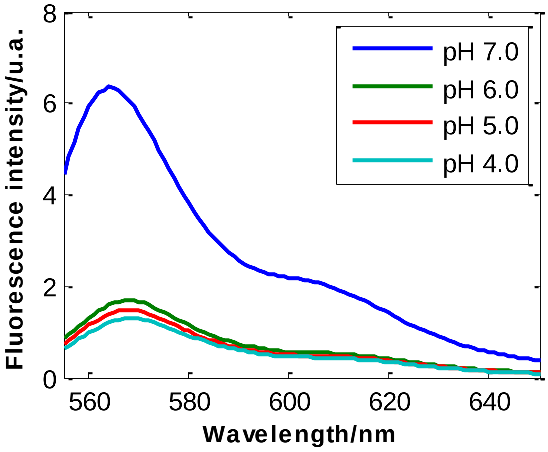
</td></tr>
</table>

F-AA-Q

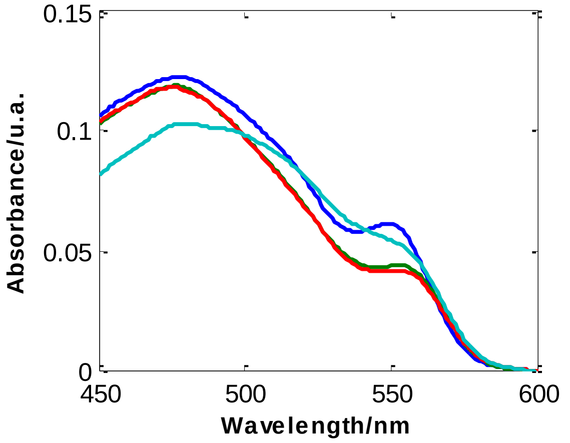

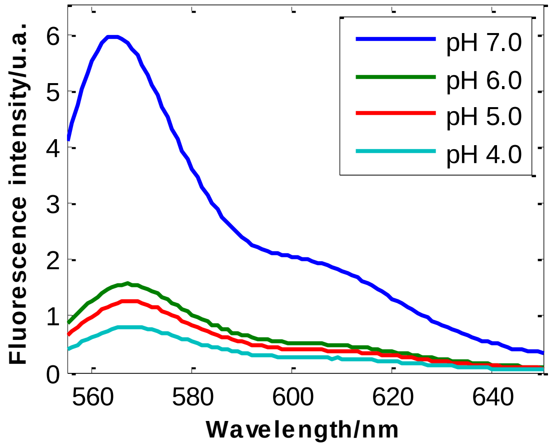

F-nmyc-Q

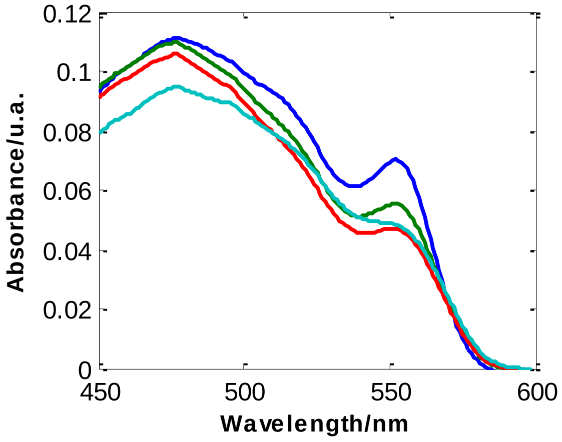

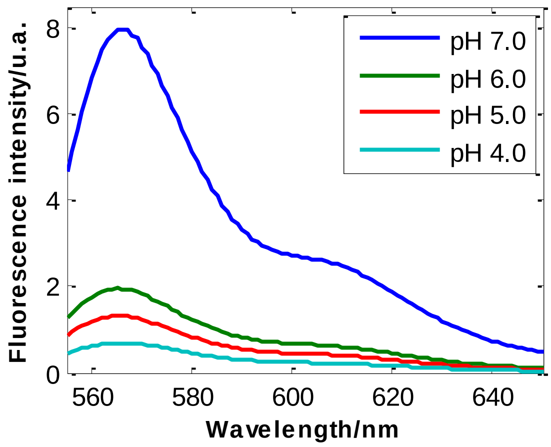

F-nmycM-Q

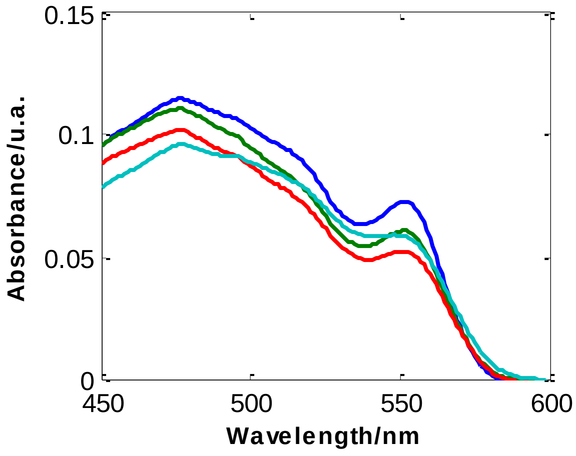

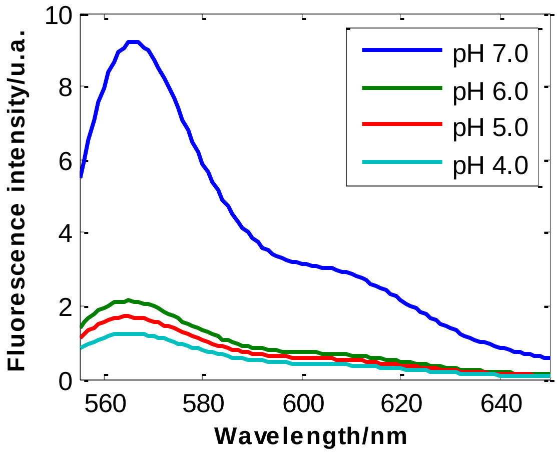

Supplementary Tables 1 and 2. Fluorescence decay fitting parameters

Supplementary Table 1. Fluorescence decay fitting parameters of F-T_{10}, F-AA-Q and F-TT-Q sequences, lifetime (τ) and the % of fractional amplitude. Experimental error : 20 ps. $: Number in parentheses is the first significant figure of error.

Sample	F-T_{10}				F-TT-Q				F-AA-Q			
**pH**	**7**	**6**	**5**	**4**	**7**	**6**	**5**	**4**	**7**	**6**	**5**	**4**
**τ_1(ns)**	1.88(8)$	1.98(1)	2.01(1)	1.94 (9)	2.35(7)	2.99(1)	3.08(1)	3.01(1)	2.38(7)	3.03(1)	3.06(1)	3.00(2)
**% Amplitude**	15.01	13.94	13.91	15.28	38.17	6.48	3.44	1.75	37.94	5.28	4.15	1.64
**τ_2(ns)**	0.71(3)	0.79(6)	0.81(7)	0.77(5)	1.11 (8)	0.89(2)	0.81(2)	0.88(1)	1.13(8)	1.02(2)	0.85(2)	0.88(2)
**% Amplitude**	44.04	42.48	42.82	42.28	44.09	5.4	2.94	2.59	43.72	4.73	3.39	2.58
**τ_3(ns)**	0.19(3)	0.24 (6)	0.25 (7)	0.23 (5)	0.35(2)	0.19(4)	0.17(3)	0.18(3)	0.34(2)	0.19(5)	0.16(3)	0.18(5)
**% Amplitude**	40.95	43.58	43.27	42.43	17.75	16.11	11.62	7.93	18.34	12.51	13.29	7.73
**τ_4(ns)**						0.04(7)	0.03(2)	0.03(2)		0.03(3)	0.03(3)	0.03(2)
**% Amplitude**						72.01	82.00	87.73		77.48	79.18	88.05

Supplementary Table 2. Fluorescence decay fitting parameters of F-nmyc-Q and F-nmycM-Q sequences, life time (τ) and the % of fractional amplitude. Experimental error : 20 ps. $: Number in parentheses is the first significant figure of error.

Sample	F-nmyc-Q				F-nmycM-Q			
**pH**	**7**	**6**	**5**	**4**	**7**	**6**	**5**	**4**
**τ_1(ns)**	2.62(5)$	2.68(1)	2.64(1)	2.75(1)	2.52(5)	2.67(7)	2.74(1)	2.63(1)
**% Amplitude**	64.06	3.79	2.98	3.39	56.00	3.4	3.09	2.61
**τ_2(ns)**	1.12(1)	0.89(1)	0.80(1)	0.95(1)	1.06(1)	0.89(1)	0.97(2)	0.81(1)
**% Amplitude**	29.39	3.13	2.79	3.86	32.63	2.73	2.93	2.92
**τ_3(ns)**	0.24(4)	0.17(4)	0.16(3)	0.19(4)	0.27(3)	0.17(3)	0.18(4)	0.16(3)
**% Amplitude**	6.56	8.81	9.69	12.1	11.38	9.85	11.23	10.96
**τ_4(ns)**		0.03(3)	0.03(2)	0.03(4)		0.03(2)	0.03(4)	0.03(2)
**% Amplitude**		84.27	84.54	80.66		84.03	82.75	83.52

Supplementary Figure 4. Curve fitting analysis

Fitting of the resolved pure decay traces obtained by MCR-ALS for the dominant conformations at the different pH values. Fitting tasks are done by using the curve fitting tool (cftool) of Matlab.

Sequence	pH 4	pH 5 and 6	pH 7
F-TT-Q	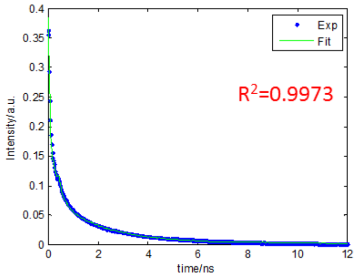	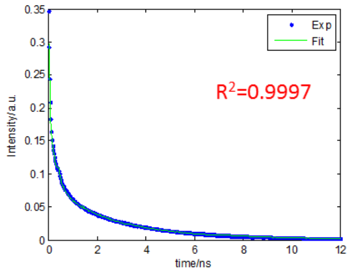	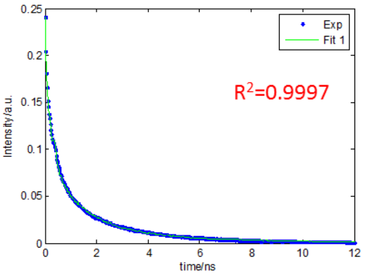
F-AA-Q	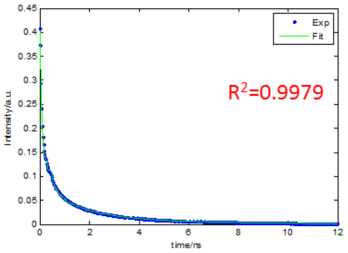	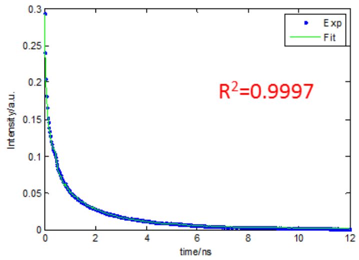	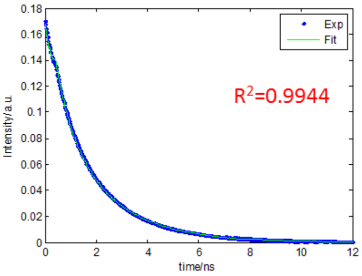

Sequence	pH 4, 5 and 6	pH 7
F-nmyc-Q	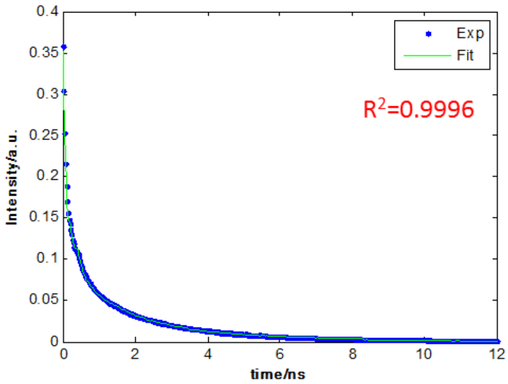	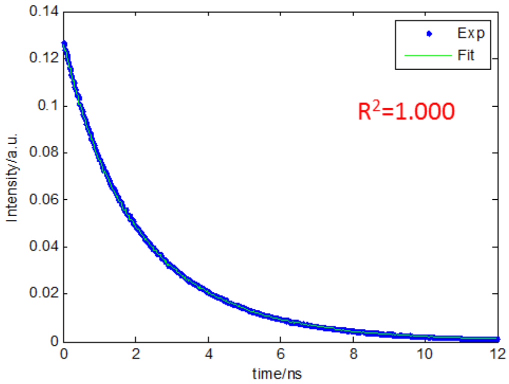
F-nmycM-Q	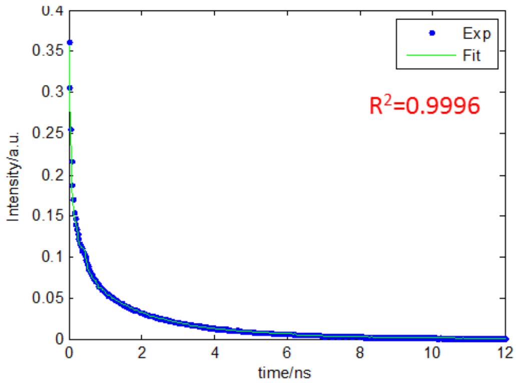	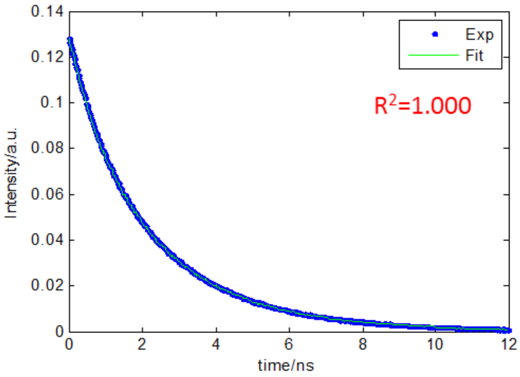

Supplementary Table 3. Comparison of fitting on pure resolved traces by MCR-ALS and on raw experimental decay curves (cftool)

Fluorescence decay fitting parameters of F-TT-Q, F-AA-Q, F-nmyc-Q and F-nmycM-Q sequences, lifetime (τ) and the % of fractional amplitude. a) Fitting of experimental decay curves and b) pure resolved traces by MCR-ALS analysis by cftool. *Pure traces that could be associated with this pH range. $: Number in parentheses is the first significant figure of error.

a) Fitting of raw experimental decay curves (cftool)

Sample	F-T10	F-TT-Q			F-AA-Q			F-nmyc-Q		F-nmycM-Q	
pH	7&6&5&4	7	6&5	4	7	6&5	4	7	6&5&4	7	6&5&4
τ_1(ns)	1.79(4)$	2.6(2)	2.90(1)	2.7(5)	2.7(1)	2.88(3)	2.7(6)	2.67(2)	2.56(1)	2.6(4)	2.5(1)
% Amplitude	33	33	23	18	32	26	18	70	25	70	22
τ_2(ns)	0.610(1)	1.2(3)	0.5(4)	0.53(1)	1.25(3)	0.59(4)	0.5(1)	1.16(3)	0.55(4)	1.06(1)	0.5(2)
% Amplitude	67	67	29	33	68	27	33	30	28	30	27
τ_3(ns)			0.07(2)	0.064(3)		0.07(5)	0.06(2)		0.066(4)		0.067(1)
% Amplitude			63	59		47	49		47		51

b) Fitting of pure resolved traces by MCR-ALS analysis (cftool)

Sample	F-TT-Q			F-AA-Q			F-nmyc-Q		F-nmycM-Q	
pH	7*	6&5*	4*	7*	6&5*	4*	7*	6&5&4*	7*	6&5&4*
τ_1(ns)	2.8 (2)$	3.08(3)	2.9(2)	2.63(3)	2.66(3)	2.8(1)	2.69(2)	2.54(4)	2.61(2)	2.52(4)
% Amplitude	25	20	14	48	18	12	68	18	69	18
τ_2(ns)	1.33(5)	0.45(1)	0.60(5)	0.46(1)	0.469(9)	0.56(4)	1.23(3)	0.49(2)	1.11(2)	0.48(2)
% Amplitude	75	28	29	52	42	28	32	30	31	28
τ_3(ns)		0.065(1)	0.089(4)		0.048(1)	0.073(3)		0.065(1)		0.066(1)
% Amplitude		66	67		40	60		52		53
